# Identification of Key Immune-Related Genes in the Progression of Septic Shock

**DOI:** 10.3389/fgene.2021.668527

**Published:** 2021-11-03

**Authors:** Jingjing Niu, Bingyu Qin, Cunzhen Wang, Chao Chen, Jianxu Yang, Huanzhang Shao

**Affiliations:** Department of Critical Care Medicine, Henan Provincial People’s Hospital, Zhengzhou University People’s Hospital, Zhengzhou, China

**Keywords:** septic shock, immune-related genes, TLR8, PPP3CA, KRAS

## Abstract

**Objective:** Septic shock is the severe complication of sepsis, with a high mortality. The inflammatory response regulates the immune status and mediates the progression of septic shock. In this study, we aim to identify the key immune-related genes (IRGs) of septic shock and explore their potential mechanism.

**Methods:** Gene expression profiles of septic shock blood samples and normal whole blood samples were retrieved from the Gene Expression Omnibus (GEO) and Genotype-Tissue Expression Portal (GTEx). The differential expression genes (DEGs) and septic shock-specific immune-related genes (SSSIRGs) were evaluated and identified, along with the immune components by “cell type identification by estimating relative subsets of RNA transcripts (CIBERSORT, version x)” algorithm. Additionally, in order to explore the key regulatory network, the relationship among SSSIRGs, upstream transcription factors (TFs), and downstream signaling pathways were also identified by Gene Set Variation Analysis (GSVA) and co-expression analysis. Moreover, the Connectivity Map (CMap) analysis was applied to find bioactive small molecules against the members of regulation network while Chromatin Immunoprecipitation sequencing (ChIP-seq) and Assay for Targeting Accessible-Chromatin with high-throughput sequencing (ATAC-seq) data were used to validate the regulation mechanism of the network.

**Results:** A total of 14,843 DEGs were found between 63 septic shock blood samples and 337 normal whole blood samples. Then, we identified septic shock-specific 839 IRGs as the intersection of DEGs and IRGs. Moreover, we uncovered the regulatory networks based on co-expression analysis and found 28 co-expression interaction pairs. In the regulation network, protein phosphatase 3, catalytic subunit, alpha isozyme (PPP3CA) may regulate late estrogen response, glycolysis and TNFα signaling *via* NFκB and HLA; Kirsten rat sarcoma viral oncogene homolog (KRAS) may be related to late estrogen response and HLA; and Toll-like receptor 8 (TLR8) may be associated with TNFα signaling *via* NFκB. And the regulation mechanisms between TFs and IRGs (TLR8, PPP3CA, and KRAS) were validated by ChIP-seq and ATAC-seq.

**Conclusion:** Our data identify three SSSIRGs (TLR8, PPP3CA, and KRAS) as candidate therapeutic targets for septic shock and provide constructed regulatory networks in septic shock to explore its potential mechanism.

## Introduction

Septic shock is the most severe complication of a severe microbial infection (sepsis), with a short treatment time-window and a high death rate (Sharawy and Lehmann, 2015). Pathogenetically, pathogens invade microorganisms; induce intracellular events in immune, epithelial, and endothelial cells (ECs); and trigger an uncontrolled systemic inflammatory response (Annane et al., 2005). In the process, the inflammatory response associated with “cytokine storm” leads to the damages to host tissues and organs, while the anti-inflammatory response reprograms the immune microenvironment and regulates the immune status (Russell and Walley, 2010). Thus, the regulation and dysregulation of immune microenvironment may play important roles in the development, progression, and prognosis of septic shock.

Current therapeutic strategies often focus on controlling the infection source by antibiotic therapy and restoring hemodynamic homeostasis by norepinephrine (Kumar, 2014). In addition, septic shock patients may also benefit from some drugs such as corticosteroids or activated protein C (Seam, 2008). Despite unceasing advances in management, septic shock still represents a major healthcare problem worldwide. Based on the crucial roles of immune microenvironment in septic shock, we suppose that targeting septic shock-specific immune-related genes (SSSIRGs) may be novel therapeutic options (Walley et al., 2014; Francois et al., 2018).

In this study, in order to identify the SSSIRGs, the public databases were searched, and the RNA profiles of blood samples in septic shock and healthy patients were downloaded. The differentially expressed SSSIRGs were identified between them. In addition, the algorithm “cell type identification by estimating relative subsets of RNA transcripts (CIBERSORT)” was used to evaluate the cell fraction. Moreover, we also constructed regulatory networks based on upstream transcription factors (TFs), SSSIRGs, immune cells, and downstream signaling pathways to decipher the potential mechanism of septic shock and identify candidate targets.

## Materials and Methods

### Data Extraction and Differential Expression Analysis

The gene expression profiles of 63 septic shock blood samples and 337 normal whole blood samples were downloaded from the Gene Expression Omnibus (GEO^1^) (Braga et al., 2019) and the Genotype-Tissue Expression Portal (GTEx, 2015^2^), respectively. The gene expression profiles were retrieved in formats of HTseq-counts and were normalized to Transcripts Per Million (TPM) based on gene length. Clinical data including demographics (age and gender) and blood collection time points (T1: within 16 h of ICU admission; T2: 48 h after study enrollment; T3: on day 7 from ICU admission or before discharge from the ICU) were also extracted from the database.

Samples and patients with incomplete clinical information were excluded, and conformers who met the inclusion and exclusion criteria were extracted for further analysis. In order to reduce the error caused by the different length and sequencing depth of each single gene in the sequencing process, firstly, raw counts (RCs) of RNA-seq data obtained from GTEx and GSE131411 were quantified, respectively, which were then standardized using voom function in limma (Linear Models for Microarray Analysis) package. To eliminate the batch effect, these two batches of RNA-seq data were corrected using the normalizeBetweenArrays function in the limma package and then merged for differential expression analysis (Smyth, 2004).

Differential expression genes (DEGs) between 63 septic shock blood samples and 337 normal whole blood samples were determined using the Edge R package (Smyth, 2004). The standard to define the DEGs was | log_2_ Fold Change (FC)| > 1.0 along with False Discovery Rate (FDR) < 0.05. Besides, the Gene Oncology (GO) and Kyoto Encyclopedia of Genes and Genomes (KEGG) items of enrichment analysis were performed to explore the biological processes (BPs), cellular components (CCs), molecular functions (MFs), and KEGG pathways that septic shock-specific DEGs enriched.

### Identification of Septic Shock-Specific Immune-Related Genes in Whole Blood

A total of 2,498 immune-related genes (IRGs) were retrieved from the ImmPort database^3^ and Molecular Signatures Database (MSigDB) v7.1,^4^ respectively (Liberzon et al., 2015). The intersection of the 2,498 IRGs and DEGs was filtered by further non-parametric test (Kruskal–Wallis *H* test) to identify SSSIRGs.

### Identification of Immune Components Potentially Regulated by Septic Shock-Specific Immune-Related Genes

The “CIBERSORT, version x” algorithm was applied to estimate the fraction of 22 types of immune cell in each whole blood sample (Newman et al., 2019). The non-parametric test (Mann–Whitney *U* test) was conducted to illuminate the different immune cell components between septic shock blood samples and normal whole blood samples. In addition, the fraction of 29 immunomodulating gene sets was quantified using single-sample Gene Set Enrichment Analysis (ssGSEA) (Barbie et al., 2009).

Correlation analysis was applied to SSSIRGs, 22 immune cells, and 29 immune gene sets. The pairwise interactions between SSSIRGs and immune cells (| correlation coefficient| > 0.500, *p*-value < 0.001) along with SSSIRGs and immune gene sets (| correlation coefficient| > 0.600, *p* < 0.001) were identified as immune components potentially regulated by SSSIRGs.

### Identification of Key Transcription Factors and Pathways of the Shock-Specific Immune-Related Genes

Fifty hallmark gene sets (signaling pathways) and the 318 TFs were retrieved from the MSigDB v7.1 (see text footnote 4) and Cistrome database,^5^ respectively (Liberzon et al., 2015; Zheng et al., 2019). Differential expression and correlation analyses were conducted to determine upstream TFs, while hallmark pathways of SSSIRGs were absolutely quantified by Gene Set Variation Analysis (GSVA) (Hanzelmann et al., 2013). Next, the correlation analysis was applied to key SSSIRGs, TFs, and aforementioned signaling pathways. Pairwise interactions between TFs and SSSIRGs (| correlation coefficient| > 0.900, *p* < 0.001), SSSIRGs, and the 50 hallmark pathways (| correlation coefficient| > 0.600, *p* < 0.001) were extracted for further analysis. All above significant interaction pairs were integrated to construct the regulation network among SSSIRGs and their upstream TFs and downstream pathways/immune cells/immune gene sets.

### Integrated Analysis of Clinical Correlation Based on Length of Hospital Stay

In order to better understand and validate the relationship between SSSIRGs and prognosis of patients with septic shock, patients were categorized into three groups (T1–T3) based on blood sample collection time points (T1: within 16 h of ICU admission; T2: 48 h after study enrollment; T3: on day 7 from ICU admission or before discharge from the ICU). Further differential expression analysis based on different time points was carried out, respectively, using the edge R package.

In addition, gene sets over-representation analysis (GSORA) was performed to evaluate the fraction of genes in tested gene clusters (Pomyen et al., 2015). In this study, 54 septic shock-related gene sets downloaded from MSigDB were divided in nine subclusters on the basis of function features, which included C1–C8 and the hallmark pathway cluster (H) (Liberzon et al., 2015). In addition, ORA was carried out to determine the enrichment level of these gene sets.^6^

Based on the key SSSIRGs obtained previously and different lengths of hospital stay (LOS) of septic shock patients, non-parametric test and clinical correlation analysis were performed, and the intersection of the results from these two analyses which were statistically significant was extracted for further analysis. Specifically, LOS served as an important sign in clinical judgment of the severity of septic shock.

### Connectivity Map Analysis

Here, we applied the Connectivity Map (CMap, build 02) to find potential inhibitors that may target key SSSIRGs. In total, 6,100 gene expression cases covering 1,309 drugs were obtained from the CMap database^7^ (Lamb et al., 2006), that is, a potential drug might correspond to various gene expression cases. Genes in each case were ranked *via* taking differential expression values between drug-untreated and drug-treated cell lines, and 6,100 gene lists associated with drugs were then generated. On the basis of identified key SSSIRGs involved in septic shock and 6,100 drug-related cases, we conducted a non-parametric test to explore the relationship between drugs and septic shock.

Information of targeting compounds is available in the mechanism of actions (MoAs)^8^ that includes transcriptional responses of various human cell lines to perturbagens, structural formulas, and various targets. On the basis of MoA, compounds which may target to septic shock-related SSSIRGs in this study were extracted.

### Validation of Chromatin Immunoprecipitation Sequencing and Assay for Targeting Accessible-Chromatin With High-Throughput Sequencing

Chromatin Immunoprecipitation sequencing (ChIP-seq) and Assay for Targeting Accessible-Chromatin with high-throughput sequencing (ATAC-seq) data were used to validated the regulation mechanism of the network. ChIP-seq and ATAC-seq data were obtained from Cistrome database (see text footnote 5) and GSE139099,^9^ respectively (Zheng et al., 2019; Grubert et al., 2020). The WashU Epigenome Browser and Gviz package were used to visualize the binding peaks (Hahne and Ivanek, 2016; Li et al., 2019).

### Processing of Single-Cell RNA Sequencing Data

Many studies supported that COVID-19 severe forms share clinical and laboratory aspects with various pathologies such as hemophagocytic lymphohistiocytosis, sepsis, or cytokine release syndrome. Fatal COVID-19 patients, as would be expected with septic shock patients, produce inflammatory reaction and cytokine storm (Bost et al., 2021). Therefore, single-cell RNA sequencing (scRNA-seq) data of peripheral blood mononuclear cells (PBMCs) from fatal COVID-19 patients were obtained from GSE150728 to determine the specific cellular location of key SSSIRGs and cell cycle regulation in this study, which can further explain the molecular pathogenesis of septic shock spatiotemporally.

The preprocessing of scRNA-seq data of seven COVID patients and six healthy samples was conducted using 10x Genomics Chromium^10^ (Chen et al., 2020). After demultiplexing, the sequencing results were divided into two paired-end reads fastq files that were then trimmed to eliminate the template switch oligo (TSO) sequence and polyA tail sequence. In addition, clean reads were aligned with the GRCh38 (Version: 100) genome assembly, which were quantified using the Cell Ranger Software (Version 1.0.0).^11^

The quantitative gene expression matrices (the row names of matrices were genes and col names were barcodes) were analyzed with Seurat pipeline (Version: 3.2.2) for further analysis (Butler et al., 2018). Only cells with less than 10% mitochondrial gene mapped and expressing more than 100,000 transcripts were extracted for further analysis. In addition, genes expressed in over three single cells were included in follow-up analyses. After the completion of quality control (QC), all samples were integrated into one Seurat object with the function of “IntegrateData,” which were then scaled and normalized with the function of “ScaleData.” The “vst” method was utilized to determine the top 1,500 variable genes. To reduce model dimensionality, principal component analysis (PCA) was initially conducted, and the top 20 PCs were incorporated as input file for Uniform Manifold Approximation and Projection for Dimension Reduction (UMAP) analysis. UMAP plots illustrating cell subclusters were constructed using the “DimPlot” and “RunUMAP” function.

### Differential Expression Analysis and Cell Type Annotation

Genes with significantly differential expression patterns from the top 1,500 variable genes were defined as DEGs with the “wilcox” method using the “FindAllMarkers” function.

To identify the cell type of each unsupervised cluster, DEGs in all subclusters were applied as potential references, which were combined with known specific cell surface biomarkers obtained from CellMarker^12^ (Zhang et al., 2019) for a comprehensive annotation of cell type. Considering the variable gene expression patterns, a specific cellular annotation method was utilized in this study. In addition, the cellular feature plots, dot plots, violin plots, and heat maps were constructed to show the marker genes of each cell type using SCANPY (Version: 1.7.1) and the Seurat R package (Version: 3.2.2) under the environment of Python 3.6 (Butler et al., 2018; Wolf et al., 2018).

### Cellular Communication Analysis

To elucidate the key cellular communication patterns and ligand-receptor pairs among various different cell types in peripheral blood, cellular communication analysis was conducted using the iTALK R package (Version: 0.1.0)^13^ (Wang et al., 2019b). Firstly, because of the scarcity of musculus resources in present mainstream cellular communication algorithms, top 1,500 variable genes were transformed into human genes using the biomaRt package (Version: 2.46.0) in a certain homologous degree with the “getLDS” function (Durinck et al., 2009). Secondly, the normalized expression matrix of these genes was incorporated into the iTALK object with the “rawParse” function. Finally, top 200 ligand-receptor pairs were shown by ligand-receptor plots and iTALK networks.

### Statistical Analysis

In this study, all statistics analysis processes were performed by the R software (version 3.6.3^14^). In descriptive statistics, mean ± SD was utilized for continuous variables in normal distribution. In addition, when encountering continuous variables in abnormal distribution, the median was utilized. A two-sided *p* < 0.05 was suggested to have statistical significance.

## Results

### Integrated Analysis of DEG and Functional Enrichment

**FIGURE 1:**
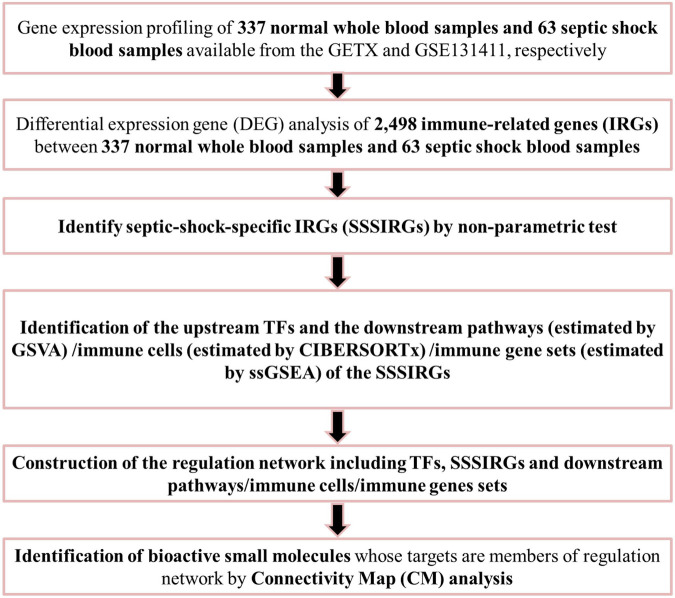
The flowchart of all analysis process.

**FIGURE 2:**
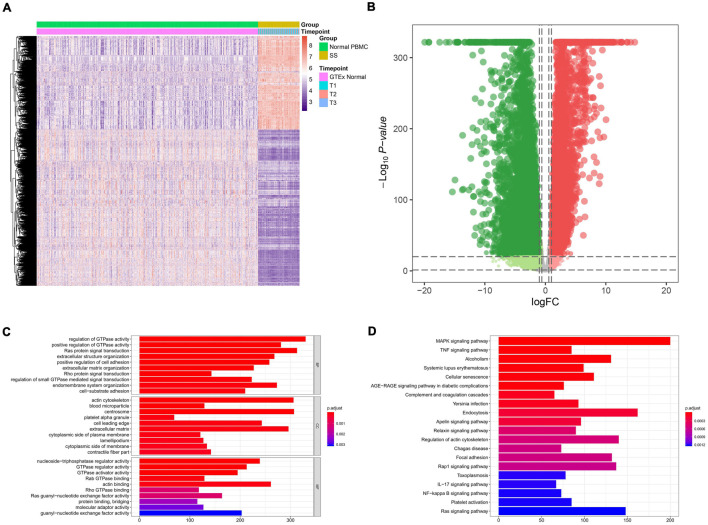
Identification of differential expression genes (DEGs) between 63 septic shock blood samples and 337 normal whole blood samples and functional annotation. **(A)** Heat map showing the expression level of DEGs. A total of 14,843 genes (9,402 down-regulated genes and 5,441 up-regulated genes) were identified as DEGs. **(B)** Volcano plot showing the differential expression patterns of DEGs, where green dots refer to down-regulated genes while red dots refer to up-regulated genes. **(C)** The output of Gene Ontology (GO) analysis. The most significant enrichment items of BPs, CCs, and MFs in GO analysis for DEGs were regulation of GTPase activity, actin cytoskeleton, nucleoside-triphosphatase regulator activity. **(D)** The output of Kyoto Encyclopedia of Genes and Genomes (KEGG) pathways enrichment analysis. The most significant KEGG pathway where DEGs mainly enriched was MAPK signaling pathway.

The flowchart of the present study is illustrated in Figure 1. A total of 14,843 genes (9,402 down-regulated genes and 5,441 up-regulated genes) were identified as DEGs between 63 septic shock blood samples and 337 normal whole blood samples (Figures 2A,B). The most important GO items of BPs, CCs, and MFs for DEGs were regulation of GTPase activity, actin cytoskeleton, and nucleoside-triphosphatase regulator activity (Figure 2C). The most significant KEGG pathway where DEGs mainly enriched was MAPK signaling pathway (Figure 2D).

### Identification of the Upstream Transcription Factors and Downstream Pathways/Immune Cells/Immune Gene Sets of Shock-Specific Immune-Related Genes

**FIGURE 3:**
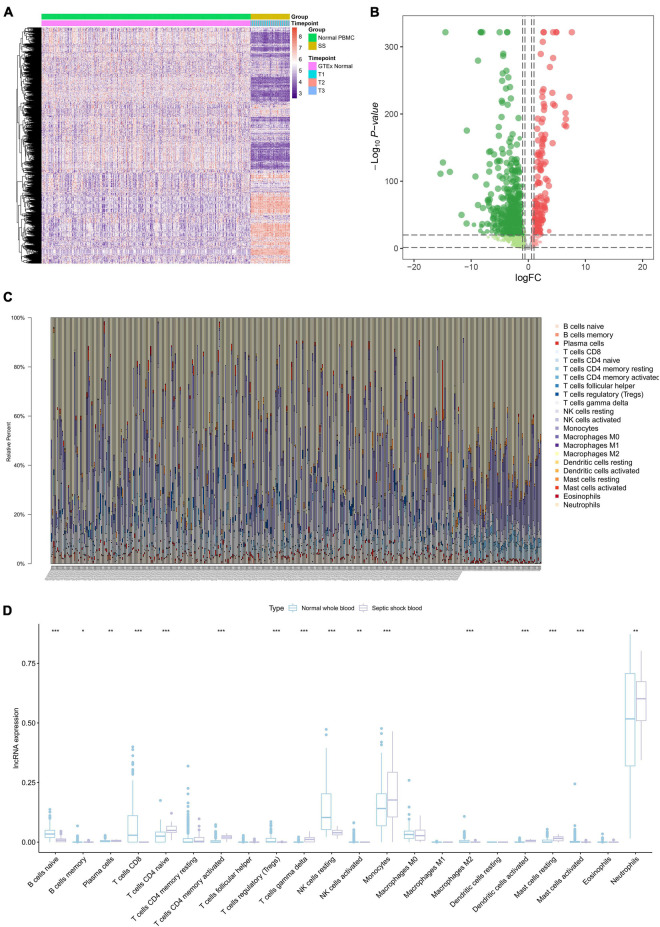
The results of differential expression genes analysis of 2,498 IRGs and CIBERSORT algorithm analysis. **(A)** Heat map showing the expression level of differentially expressed septic shock-specific immune-related genes (SSSIRGs). A total of 839 SSSIRGs (666 down-regulated genes and 173 up-regulated genes) were identified as the intersection of 14,843 DEGs and 2,498 IRGs in septic shock peripheral blood. **(B)** Volcano plot showing the differential expression patterns of SSSIRGs, where green dots refer to down-regulated genes while red dots refer to up-regulated genes. **(C)** Bar chart showing the percentage of 22 kinds of immune cells in each sample by estimating relative subsets of RNA transcripts (CIBERSORT). **(D)** Box plot showing the fraction of 22 immune cells in normal whole blood and septic shock blood by Mann–Whitney *U* test. The results suggested that B cells naïve (*p* < 0.001), B cells memory (*p* < 0.050), Plasma cells (*p* < 0.010), T cells CD8 (*p* < 0.001), T cells CD4 naïve (*p* < 0.001), T cells CD4 memory activated (*p* < 0.001), T cells regulatory (Tregs) (*p* < 0.001), T cells gamma delta (*p* < 0.001), NK cells resting (*p* < 0.001), NK cells activated (*p* < 0.010), Monocytes (*p* < 0.001), Macrophages M2 (*p* < 0.001), Dendritic cells activated (*p* < 0.001), Mast cells resting (*p* < 0.001), Mast cells activated (*p* < 0.001), and Neutrophils (*p* < 0.010) were differentially enriching between septic shock and normal whole blood samples. *p < 0.05, **p < 0.01, ***p < 0.001.

A total of 839 SSSIRGs (666 down-regulated genes and 173 up-regulated genes) were extracted from the intersection of 14,843 DEGs and 2,498 IRGs in septic shock blood, and the expression level of these SSSIRGs were shown in the heat map (Figure 3A) and volcano plot (Figure 3B).

The CIBERSORT algorithm was used to estimate the fraction of 22 types of immune cell in each blood sample (Figure 3C). The results of the Mann–Whitney *U* test suggested that B cells naïve (*p* < 0.001), B cells memory (*p* < 0.050), Plasma cells (*p* < 0.010), T cells CD8 (*p* < 0.001), T cells CD4 naïve (*p* < 0.001), T cells CD4 memory activated (*p* < 0.001), T cells regulatory (Tregs) (*p* < 0.001), T cells gamma delta (*p* < 0.001), NK cells resting (*p* < 0.001), NK cells activated (*p* < 0.010), Monocytes (*p* < 0.001), Mast cells activated (*p* < 0.001), Dendritic cells activated (*p* < 0.001), Mast cells resting (*p* < 0.001), Macrophages M2 (*p* < 0.001), and Neutrophils (*p* < 0.010) were differentially enriched between the septic shock and normal whole blood samples (Figure 3D).

**FIGURE 4:**
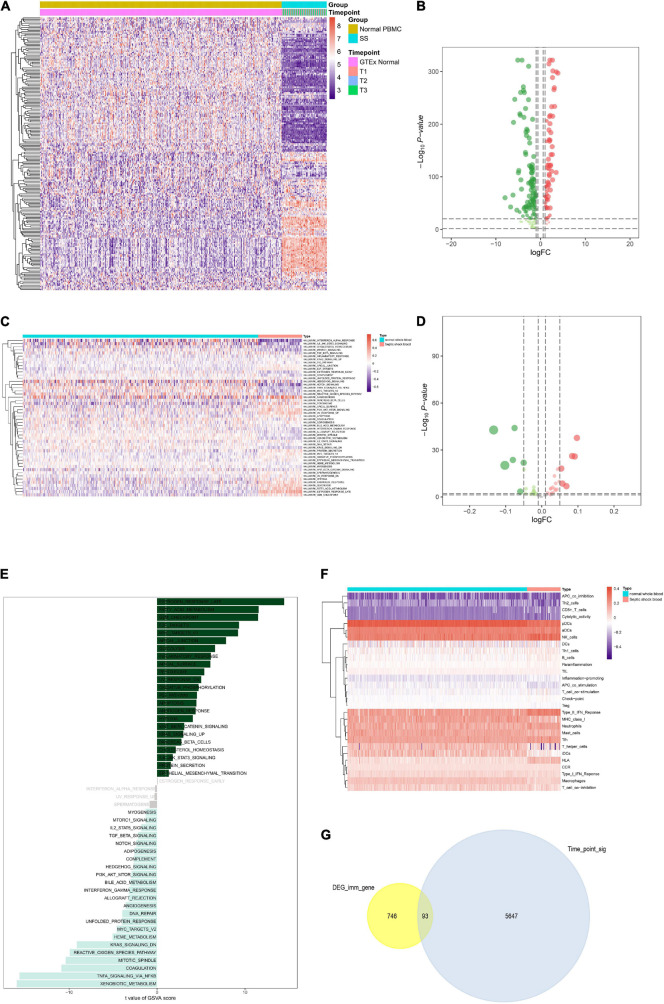
Identification of upstream TFs and the downstream pathways/immune cells/immune gene sets of SSSIRGs. **(A)** Heat map showing the expression level of differentially expressed TFs. **(B)** Volcano plot showing the differential expression patterns of TFs, where green dots refer to down-regulated genes while red dots refer to up-regulated genes. **(C)** Heat map showing the expression level of hallmark signaling pathways in normal whole blood and septic shock blood by Gene Set Variation Analysis (GSVA). **(D)** Volcano plot showing the differential expression patterns of hallmark signaling pathways, where green dots refer to down-regulated pathways while red dots refer to up-regulated pathways. **(E)** The bar plot revealing the *t*-value of GSVA score of hallmark pathways. **(F)** Heat map showing the expression level of 29 immune gene sets *via* single-sample Gene-Set Enrichment Analysis (ssGSEA). **(G)** Venn plot showing that only 93 of 839 SSSIRGs with differentially expressed patterns were significantly associated with blood collection time points (T1–T3).

Furthermore, heat map and volcano plot were used to illustrate the DEG analysis results of the TFs (Figures 4A,B). Absolute quantification of hallmark pathways was illustrated by the heat map as well as volcano plot (Figures 4C,D). In addition, the *t*-score of each signaling pathway was shown in the bar plot (Figure 4E). Further, expression levels of 29 immune gene sets in normal whole blood and septic shock blood acquired by ssGSEA were displayed in the heat map (Figure 4F). Only 93 of 839 IRGs were not only differentially expressed between septic shock and normal whole blood samples but also significantly associated with blood collection time points (T1–T3). Thus, they were defined as the SSSIRGs (Figure 4G).

**FIGURE 5:**
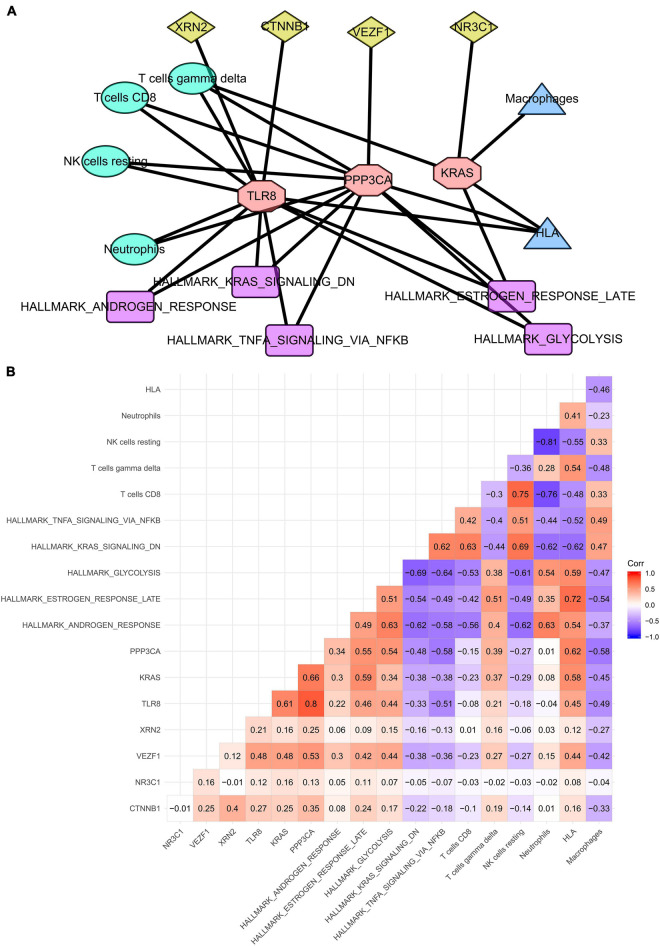
Construction of regulation network among key SSSIRGs and their upstream TFs and the downstream pathways/immune cells/immune gene sets in septic shock. **(A)** Overview of the protein–protein interaction network. Three SSSIRGs (TLR8, PPP3CA, and KRAS) were screened by the co-expression analysis with 28 co-expression interaction pairs including four TF-SSSIRG interaction pairs, nine SSSIRG-immune-cell interaction pairs, four SSSIRG-immune-gene-set interaction pairs, and 11 SSSIRG-pathway interaction pairs used for construction of the regulation network. **(B)** Co-expression heat map illuminating all co-expression patterns of SSSIRGs with both upstream TFs and the downstream signaling pathways/immune cells/immune gene sets.

Finally, three SSSIRGs Toll-like receptor 8 [TLR8, protein phosphatase 3, catalytic subunit, alpha isozyme (PPP3CA), and Kirsten rat sarcoma viral oncogene homolog (KRAS)] were screened by the co-expression analysis with 28 co-expression interaction pairs including four TF-SSSIRG interaction pairs, nine SSSIRG-immune-cell interaction pairs, four SSSIRG-immune-gene-set interaction pairs, and 11 SSSIRG-pathway interaction pairs (Figure 5A), based on which a regulation network was constructed. Besides, all correlation patterns of SSSIRGs with both the upstream TFs and the downstream signaling pathways/immune cells/immune gene sets were illustrated by a heat map (Figure 5B).

Importantly, vascular endothelial zinc finger 1 (VEZF1) may upregulate the expression level of PPP3CA (*R* = 0.530, *p* < 0.001), which may further increase the activities of T cells gamma delta (*R* = 0.390, *p* < 0.001) and HLA (*R* = 0.620, *p* < 0.001) while suppressing the activity of hallmark TNFα signaling *via* NFκB (*R* = −0.580, *p* < 0.001); VEZF1 may enhance the expression of KRAS (*R* = 0.480, *p* < 0.001), which may further activate the activities of hallmark late estrogen response (*R* = 0.590, *p* < 0.001), T cells gamma delta (*R* = 0.370, *p* < 0.001), and HLA (*R* = 0.580, *p* < 0.001); VEZF1 may stimulate the expression of TLR8 (*R* = 0.480, *p* < 0.001), which may inhibit the activities of hallmark TNFα signaling *via* NFκB (*R* = −0.510, *p* < 0.001) and macrophages (*R* = −0.490, *p* < 0.001) while increasing the activity of T cells gamma delta (*R* = 0.210, *p* < 0.001).

### Integrated Analysis of Clinical Correlation and Connectivity Map Analysis

**FIGURE 6:**
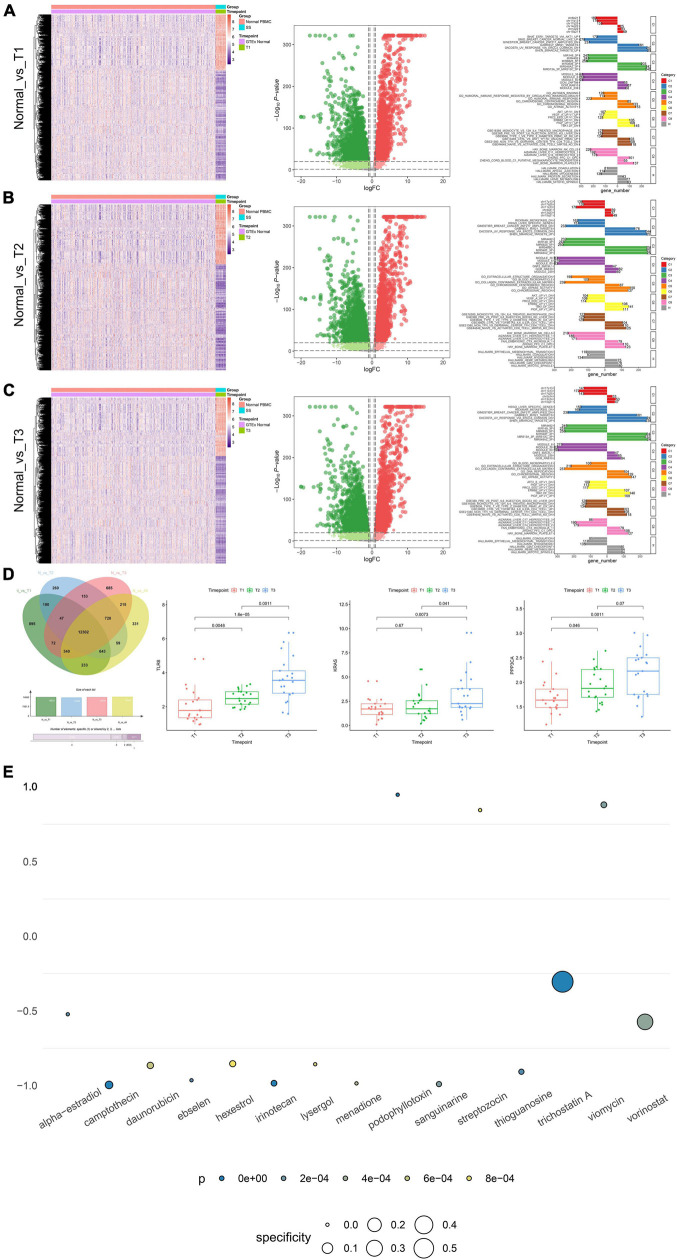
The results of clinical correlation analysis and CMap analysis. **(A–C)** Significant gene differential expression patterns of the key SSSIRGs identified previously (TLR8, KRAS, and PPP3CA) between normal samples and septic shock patients in different time point groups (T1–T3) are illustrated in the heat maps and volcano plots, respectively; the bar plot showing the DEGs belongs to several gene sets of immune response and inflammation, including immune response mediated by circulating immunoglobulin, humoral immune response gene set, and hallmark coagulation. **(D)** Venn plot showing 12,302 DEGs that were significantly differentially expressed between normal samples and all time point groups; the box plots of non-parametric test (Kruskal–Wallis *H* test) showing the three identified key SSSIRGs (TLR8, KRAS, and PPP3CA) were significantly associated with the length of hospital stay (LOS) (*p* < 0.050). **(E)** Dot plot showing 15 potential bioactive small molecules targeting to SSSIRGs including alpha-estradiol, camptothecin, daunorubicin, ebselen, hexestrol, irinotecan, lysergol, menadione, podophyllotoxin, sanguinarine, streptozocin, thioguanosine, trichostatin A, and viomycin and vorinostat (*p* < 0.001). Importantly, podophyllotoxin with the highest specificity was considered as the best compound targeting to key SSSIRGs.

Significantly, gene differential expression patterns between normal samples and septic shock patients in different time point groups (T1–T3) are illustrated by the heat maps and volcano plots, respectively (Figures 6A–C). Importantly, the key SSSIRGs identified previously (TLR8, KRAS, and PPP3CA) were all significantly differentially expressed in the analysis of three time point groups, which was consistent with the previous results. Additionally, the ORA suggested that the DEGs were belonging to several gene sets of immune response and inflammation, including immune response mediated by circulating immunoglobulin, humoral immune response gene set, and hallmark coagulation (Figures 6A–C).

The Venn plot showed there were 12,302 DEGs that were significantly differential expressed between normal samples and all time point groups. The box plots of non-parametric test (Kruskal–Wallis *H* test) showed that the three identified key SSSIRGs (TLR8, KRAS, and PPP3CA) were significantly related to the length of hospital stay (LOS) (*p* < 0.050) (Figure 6D). Besides, the CMap analysis suggested that 15 bioactive small molecules including alpha-estradiol, camptothecin, daunorubicin, ebselen, hexestrol, irinotecan, lysergol, menadione, podophyllotoxin, sanguinarine, streptozocin, thioguanosine, trichostatin A, viomycin, and vorinostat were identified as significant inhibitors (*p* < 0.001). Importantly, podophyllotoxin with the highest specificity was considered as the best compound targeting to key SSSIRGs (Figure 6E).

### Validation of Chromatin Immunoprecipitation Sequencing and Assay for Targeting Accessible-Chromatin With High-Throughput Sequencing

**FIGURE 7:**
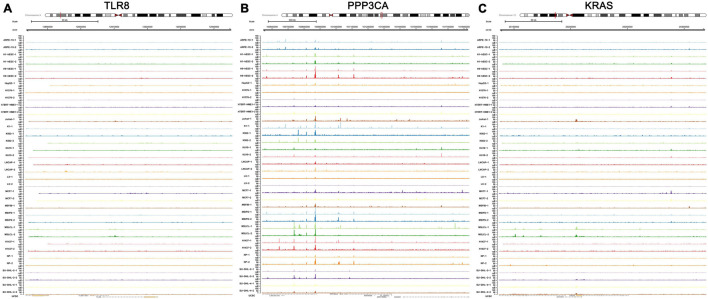
Validation of three key IRGs (TLR8, PPP3CA, and KRAS) in ATAC-seq data available from GSE139099. Binding peaks of three key IRGs [TLR8 **(A)**, PPP3CA **(B)**, and KRAS **(C)**] in ATAC-seq data of 24 different cell lines from a variety of tissues were visualized by Gviz.

**FIGURE 8:**
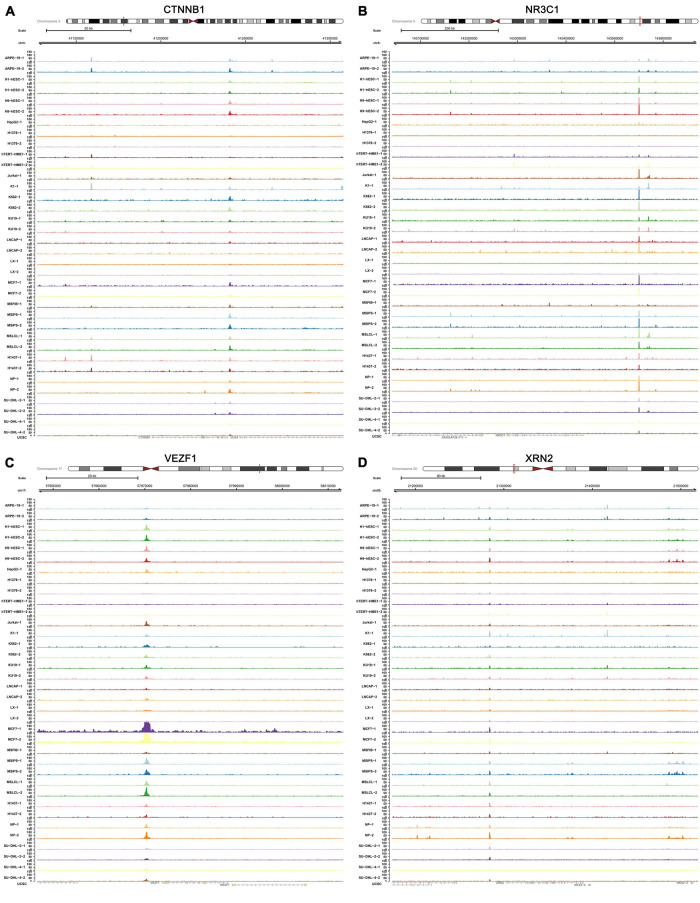
Validation of four key TFs (CTNNB1, NR3C1, VEZF1, and XRN2) in ATAC-seq data available from GSE139099. Binding peaks of three key four key TFs [CTNNB1 **(A)**, NR3C1 **(B)**, VEZF1 **(C)**, and XRN2 **(D)**] in ATAC-seq data of 24 different cell lines from a variety of tissues were visualized by Gviz.

**FIGURE 9:**
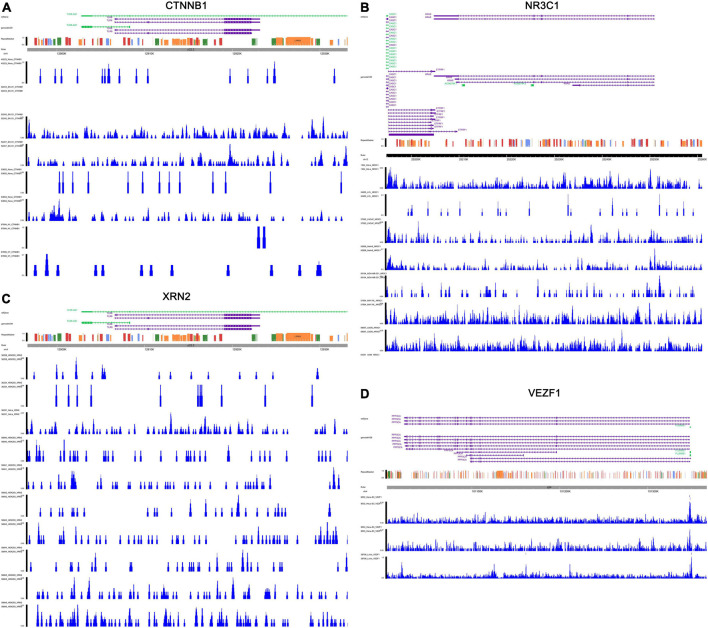
Validation of the transcriptional regulation mechanisms of four TF-IRG pairs in ChIP-seq data available from Cistrome database. The transcriptional regulation mechanisms of four TF-IRG pairs [CTNNB1-TLR8 **(A)**, NR3C1-PPP3CA **(B)**, XRN2-TLR8 **(C)**, and VEZF1-KRAS **(D)**] were validated by ChIP-seq data available from Cistrome database.

The transcriptional regulation mechanisms between four key TFs (CTNNB1, XRN2, VEZF1, and NR3C1) and three key IRGs (TLR8, PPP3CA, and KRAS) were verified using ChIP-seq and ATAC-seq data. Binding peaks of three key IRGs and four key TFs in ATAC-seq data of 24 different cell lines from a variety of tissues are shown in Figures 7, 8, respectively. Additionally, transcriptional regulation mechanisms of four TF-IRG pairs were validated by ChIP-seq data available from the Cistrome database (Figure 9).

### Integrated Analysis of Single-Cell RNA Sequencing

**FIGURE 10:**
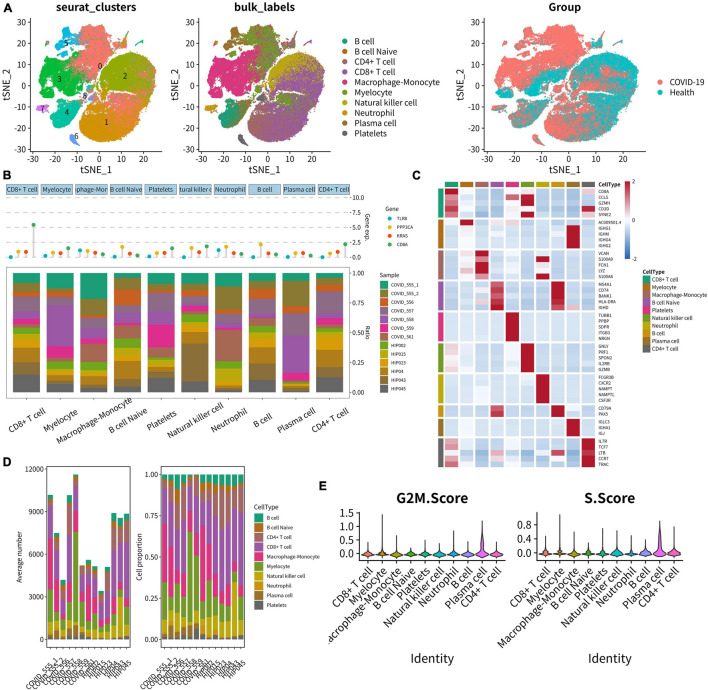
Integrated analysis of scRNA-seq. **(A)** UMAP scatter plots showing 10 main cell types using unsupervised clustering. **(B)** Cleveland’s dot plot showing the expression levels of key SSSIRGs, which were TLR8, PPP3CA, KRAS, and CD8A; the bar plot shows the proportions of 10 cell types in each sample. **(C)** Heat map illustrating the expression levels of the top 5 marker genes in 10 cell clusters. **(D)** Bar plot illustrating the fractions of these cell types in every sample. **(E)** Violin plot showing the majority of these cells was in the G2M and S phases.

In total, 10 main cell types were identified using the UMAP analysis of unsupervised clustering (Figure 10A). DEGs from the top 1,500 variable genes were identified in all single cells. Cleveland’s dot plot was used to show the expression levels of key SSSIRGs, which were TLR8, PPP3CA, KRAS, and CD8A (Figure 10B). In addition, the proportions of 10 cell types identified previously in each sample were illustrated by the bar plot (Figure 10B). The heat map illustrated expression levels of the top 5 markers in 10 aforementioned subclusters (Figure 10C). Further, the bar plot was constructed to illustrate the fractions of these cell types (Figure 10D). Moreover, analysis of cell cycle demonstrated that the major cell types were in the G2M and S phases (Figure 10E).

**FIGURE 11:**
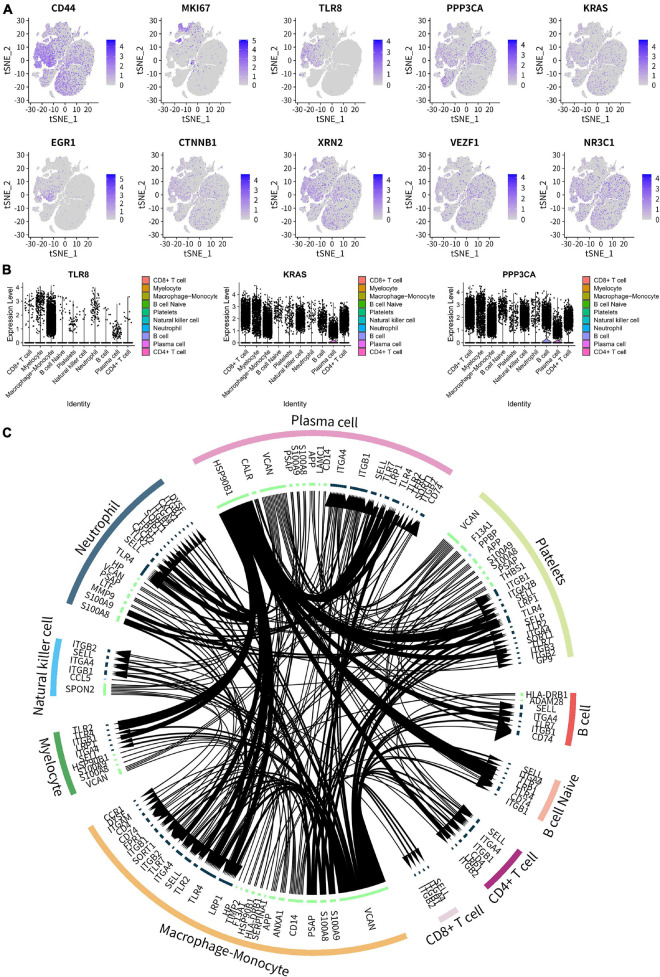
Cell type annotation and cellular communication analysis. **(A)** Cellular feature plots showing expression levels and specific cellular locations of the key SSIRGs (CD44, MKI67, TLR8, PPP3CA, and KRAS) and TFs (EGR1, CTNNB1, XRN2, VEZF1, and NR3C1) in different immune cells. **(B)** Violin plot showing the expression levels of three key SSSIRGs (TLR8, KRAS, and PPP3CA) in 10 immune cell clusters. TLR8 was significantly highly expressed in Macrophage–Monocyte, Myelocyte, and Neutrophil; KRAS was significantly highly expressed in Myelocyte, CD8+ T cell, and Macrophage–Monocyte; PPP3CA was significantly highly expressed in CD8+ T cell, Myelocyte, and Macrophage–Monocyte. **(C)** Intersected cellular communication ligand-receptor plots showing the specific mechanism of intercellular signal transduction.

The cellular feature plots of key SSIRGs (CD44, MKI67, TLR8, PPP3CA, and KRAS) and TFs (EGR1, CTNNB1, XRN2, VEZF1, and NR3C1) were used to show the expression levels and specific cellular locations of these key biomarkers in different immune cells (Figure 11A). In addition, Figure 11B illustrates the expression levels of three key SSSIRGs (TLR8, KRAS, and PPP3CA) in 10 immune cell clusters. Importantly, TLR8 was significantly highly expressed in Macrophage–Monocyte, Myelocyte, and Neutrophil; KRAS was significantly highly expressed in Myelocyte, CD8+ T cell, and Macrophage–Monocyte; PPP3CA was significantly highly expressed in CD8+ T cell, Myelocyte, and Macrophage–Monocyte (Figure 11B). Intersected cellular communication ligand-receptor plots showed the specific mechanism of intercellular signal transduction. Importantly, there was significantly strong intercellular communication among these cell clusters (Figure 11C).

## Discussion

Septic shock is a life-threatening disease associated with a dysregulated immune response to infections, subsequently inducing tissue and organ injuries (Esposito et al., 2017). Although immune microenvironment is supposed to participate in the development of septic shock, its immune-related biomarkers and targets, however, have not been explored. In this study, we identified three key SSSIRGs (TLR8, PPP3CA, and KRAS) *via* bioinformatics methods. In addition, by the constructed regulatory network, we also found the potential relationship between SSSIRGs (TLR8, PPP3CA, and KRAS) and signaling pathways (late estrogen response, glycolysis, and TNFα signaling *via* NFκB)/immune gene sets (HLA).

In septic shock, immune cells, such as neutrophils, macrophages, lymphocytes, and dendritic cells, take part in the development of inflammatory response and the patient’s inflammatory state (adaptive immunosuppression) (Stiel et al., 2018). Neutrophils secrete pro-inflammatory mediators and cytokines (IL-1β, IL-6, IL-12, and IL-17) to induce T-cell differentiation and stimulate the microbicidal activity of macrophages (Ferguson et al., 1999; Qiu et al., 2019). Septic shock is also associated with an activation cascade of cytokine production (cytokine storm), which contributes to severe tissue damage (Chousterman et al., 2017). Despite the key pathophysiological role of cytokines during infection, no specific treatment targeting inflammation was shown to be effective in sepsis shock, neither were antibodies targeting TNFα, IL-1, and LPS (Opal et al., 2013; Payen et al., 2015). Thus, identification of the SSSIRGs, along with their regulatory networks, may provide candidate therapeutic targets for septic shock.

In this study, based on a comprehensive bioinformatic analysis, differentially expressed TFs, SSSIRGs, downstream hallmark pathways/immune-related pathways, and immune cells were all identified, which were used to construct a regulatory network where SSSIRGs occupied the communications center, providing the most significant candidate predictors and therapeutic targets for septic shock. Specifically, mediated by VEZF1, upregulation of PPP3CA (*R* = 0.530, *p* < 0.001) may further activate T cells gamma delta (*R* = 0.390, *p* < 0.001) and HLA (*R* = 0.620, *p* < 0.001) while suppressing hallmark TNFα signaling *via* NFκB (*R* = −0.580, *p* < 0.001); VEZF1 may also enhance the expression level of KRAS (*R* = 0.480, *p* < 0.001), further activating hallmark late estrogen response (*R* = 0.590, *p* < 0.001), T cells gamma delta (*R* = 0.370, *p* < 0.001), and HLA (*R* = 0.580, *p* < 0.001); induced by VEZF1, upregulation of TLR8 (*R* = 0.480, *p* < 0.001) was implicated in the inhibition of hallmark TNFα signaling *via* NFκB (*R* = −0.510, *p* < 0.001) and macrophages (*R* = −0.490, *p* < 0.001) and the activation of T cells gamma delta (*R* = 0.210, *p* < 0.001). Moreover, podophyllotoxin was considered as the best small-molecular inhibitor targeting key SSSIRGs in this study with the highest specificity (*p* < 0.001). Significant SSSIRG regulatory mechanisms were further determined using CHIP-seq and ATAC-seq validation, and the specific cellular location of key TFs and SSSIRGs was determined by scRNA-seq validation.

Vascular endothelial zinc finger 1 is the key TF identified in this study, encoding a zinc finger TF that is critical in lymphangiogenesis and developmental angiogenesis. As a TF expressed in ECs at the angiogenesis site, down-regulation of VEZF1 may induce the exhibition of vascular remodeling, loss of vascular integrity, internal bleeding, and embryonic death (Xiong et al., 1999; Miyashita et al., 2004). Importantly, dysfunction of endothelial barrier refers to a hallmark of septic shock, which is partly mediated *via* pathways regulating the endothelial barrier assembly in angiogenesis (Faiotto et al., 2017). Not surprisingly, enhanced expression levels of significant angiogenic biomarkers including angiopoietin-2 and VEGF-A have been demonstrated in septic shock, while the role of VEZF1 in sepsis is rarely reported (Luz Fiusa et al., 2013). Hence, VEZF1 may be implicated in the regulation of angiogenesis in septic shock, which is one of the novel targets of anti-sepsis drug exploration. Importantly, angiogenesis inhibitors have attracted much attention as anti-inflammatory agents for chemotherapy. For instance, a non-cytotoxic low dose of paclitaxel could effectively suppress angiogenesis elicited by inflammation and alleviate septic shock-induced acute lung injury (Belotti et al., 1996; Wang et al., 2019a). Nevertheless, direct interaction between VEZF1 and the key SSSIRGs in this study is still a black box. Further studies to elaborate on the regulatory mechanism are warranted.

Toll-like receptor 8 is a key member of TLR family, which comprises pattern recognition receptors in sensing microbial invasion and initiating innate and adaptive immune responses *via* stimulating bactericidal activities of leukocytes and inducing the maturation of antigen presenting cells (Netea et al., 2004; Savva and Roger, 2013). It is the major endosomal sensor of degraded RNA in human monocytes and macrophages (Ehrnström et al., 2017). In addition, TLR8 can also crosstalk with other innate receptors and sense microbial nucleic acids, especially the single-stranded RNA (ssRNA), in infection and immunity (Lien and Ingalls, 2002; Kawai and Akira, 2011). Potent anti-viral immune responses can be induced by TLR8 triggering, which is featured by the generation of interferons (IFNs) and NF-κB-related cytokines. TLR8 agonists are mainly utilized to treat viral diseases, as well as adjuvants for malignancy and vaccines for infectious disease (Savva and Roger, 2013). In this study, we also found that TLR8 may play an important role by regulating TNFα signaling *via* NFκB. Moreover, based on the previous studies, the role of TLR8 in sensing viral ssRNA as a danger signal may result in the induction of Type I IFN as well as the pro-inflammatory cytokine, TNFα (Pang et al., 2011). Thus, we suppose that TLR8 may take part in the progression of septic shock by TNFα signaling *via* NFκB.

Due to the roles of TLR8 induced by viruses or bacteria, it is of interest to generate the antagonist of TLR8 to counteract the overwhelming immune activation and treat the infection. Although synthetic antagonists of nucleic acid-sensing TLRs have been developed and some can inhibit the human TLR8 response to ssRNA (E6446, CL097, isatoribine, R-848, and GS-9620), their clinical outcomes are not favorable (Franklin et al., 2011; Savva and Roger, 2013). Thus, there is still a pressing need to explore effective TLR8 antagonists and verify them by preclinical experiments and clinical trials.

Protein phosphatase 3, catalytic subunit, alpha isozyme encodes a calcineurin protein, which is an isoform of a subunit of Ca2+ interacting serine/threonine phosphatase. It can be involved in synaptic vesicle recycling and take part in the calcium-dependent dephosphorylation of dynamin-1 (Myers et al., 2017). In chronic infections, calcineurin is associated with the sustainability of exhausted CD8+ T cells (Khan et al., 2019). In addition, calcineurin-NFAT signaling can regulate the function of myeloid leukocytes during immune responses to bacteria, parasitic infections, and viruses (Ranjan et al., 2014; Bendickova et al., 2017).

Importantly, hypoperfusion induced by septic shock results in cellular hypoxia and mitochondrial dysfunction, eventually causing multi-organ failure (Cecconi et al., 2018). Hypoxia induces gene expression reprogramming which can facilitate anaerobic generation of energy, increased oxygen transport by activation of angiogenesis, erythropoiesis, and various other adaptive cellular changes allowing survival under circumstances of low oxygen in septic shock. Hypoxia-inducible factors (HIFs) are critical regulators involved in this adaptive response (Semenza, 2012), the activity of which can be inhibited by PPP3CA, hence playing an important part in the transcriptional response to cellular hypoxia induced by septic shock (Karagiota et al., 2019). Specific inhibitors of PPP3CA may deactivate this vicious cycle, achieving an adequate cellular oxygen transport and functional recovery.

In the regulation network of this study, we found that PPP3CA might regulate late estrogen response, glycolysis, and TNFα signaling *via* NFκB and HLA. As such, the calcineurin-NFAT pathway may cooperate with NFκB to modulate gene expression on antimicrobial immune responses (Bronk et al., 2014). Based on the roles of calcineurin in the infection, the inhibition of calcineurin-NFAT pathway is used in transplant medicine and reported to suppress T-cell responses (Yeh and Markmann, 2013). Moreover, the calcineurin inhibitors such as tacrolimus and peptide 11R-VIVIT can reduce the expression of IL-12p40, IL-12p70, and IL-23 and mediate macrophage function in murine colitis (Elloumi et al., 2012). Thus, targeting calcineurin may also be a treatment option for septic shock.

Kirsten rat sarcoma viral oncogene homolog, the most commonly mutated oncogene in human cancer, functions as an on/off switch in cellular communication and proliferation. In addition, KRAS is a RAS isoform along with HRAS and NRAS (Wu et al., 2019). During infection, Ras and Rap1 are also crucial for host innate immune defenses (Jang et al., 2018). As for KRAS, KRAS silencing increases the production of IL-4, indicating that KRAS is associated with TH1 response. In addition, KRAS is found to mediate the Ova-specific T-reg cells and macrophages to regulate antigen-specific immune response (Jha et al., 2020). A recent study also reported that KRAS is implicated in the neonatal sepsis based on a comprehensive bioinformatics analysis (Meng et al., 2018). Thus, our results indicate that KRAS may also participate in the progression of septic shock and be regarded as a potential therapeutic target, providing a new enlightenment for sepsis research.

In the present study, based on CMap analysis, podophyllotoxin was suggested as the best inhibitor targeting to key SSSIRGs with the highest specificity (*p* < 0.001). Podophyllotoxin is one of the newly synthesized anti-tumor agents, which has been demonstrated to have a wide spectrum of cytotoxic activity against a large number of human cancer cell lines such as multidrug-resistant cell lines (Gan et al., 1994). The human peripheral blood-derived monocytes (PBMs) play an important role in the immune system *via* the antigen presenting cells, the secretion of multiple cytokines, and their participation during inflammation responses (Klostergaard et al., 1986). Current researches report that podophyllotoxin is also endowed with selective inhibitory effects of several cytokines generated by activated PBM, such as TNFα and IL-113. In addition, podophyllotoxin can also rapidly inhibit cytokine secretion by PBM after their activation *via* multiple stimuli including LPS and IFN-γ, whereas currently no specific treatment targeting inflammation was effective in sepsis shock, neither were antibodies targeting TNFα, IL-1, and LPS (Gan et al., 1994; Opal et al., 2013; Payen et al., 2015). Taken together, the dual effects of podophyllotoxin as an anti-cancer agent and immunomodulator are of critical therapeutic significance in some disease manifestations, including tumor, inflammation responses, and septic shock.

There were still several limitations to the present study. First, the data were downloaded from public sources that were statistically imperfect with limited samples. Second, partial information of other confounding variables was not available in this study. Third, a prospective study is needed to evaluate the significance of these key SSSIRGs in terms of long-term clinical outcomes and possible applications of molecular drugs for septic shock. Finally, further experiment is an absolute necessity in demonstrating the regulatory mechanisms of key SSSIRGs implicated in septic shock. Therefore, CHIP-seq data of the key TFs and SSSIRGs in this study were obtained and analyzed, which broadened the scope of validation and supplemented the specific regulatory mechanisms of SSSIRG action involved in pathogenesis of septic shock. ATAC-seq data of the aforementioned key biomarkers were also applied to validate the SSSIRG regulatory mechanisms. Moreover, the cell subtype locations of the key SSSIRGs and TFs were determined *via* scRNA-seq validation. Additionally, a comprehensive transcriptome bioinformatics analysis of spatial transcriptome and scRNA-seq, fluorescence immunohistochemistry, and SSSIRG-related direct mechanism experiments would be the further research directions.

## Conclusion

The current study firstly decoded the molecular pathogenesis of septic shock *via* comprehensive bioinformatics analysis and validation spatiotemporally. The specific cellular subtypes, TFs, key SSSIRGs (TLR8, PPP3CA, and KRAS), key cellular communication interactions, immune cells, and signaling pathways were identified, providing the most significant candidate predictors and therapeutic targets for septic shock in cellular and molecular levels. Notably, we concluded that the SSSIRGs were obviously differentially expressed in septic shock at different time points. Interestingly, SSSIRGs occupied the communications center of the whole regulation process, providing the most significant candidate predictors and therapeutic targets for septic shock. Moreover, this study also provided candidate small-molecule compounds as potential targets for the treatment of septic shock. Future researches can focus on the spatiotemporally fine regulation mechanism and prospective clinical studies to validate the significance of these key SSSIRGs in terms of long-term clinical outcomes and potential applications of these molecular and cellular targets for septic shock therapy.

## Data Availability Statement

Publicly available datasets were analyzed in this study. This data can be found here: the datasets generated and/or analyzed during the current study are available in the Gene Expression Omnibus (GEO, https://www.ncbi.nlm.nih.gov/geo/query/acc.cgi?acc=GSE131411) and the Genotype-Tissue Expression Portal (GTEx, https://commonfund.nih.gov/GTEx/). ChIP-seq and ATAC-seq data were obtained from Cistrome database (http://cistrome.org/) and GSE139099 (https://www.ncbi.nlm.nih.gov/geo/query/acc.cgi?acc=GSE139099), respectively. Single-cell RNA sequencing (scRNA-seq) data of peripheral blood mononuclear cells (PBMCs) from fatal COVID-19 patients was obtained from GSE150728.

## Ethics Statement

The study was approved by the Ethics Committee of Zhengzhou University People’s Hospital.

## Author Contributions

JN, BQ, CW, CC, JY, and HS: conception and design, collection and assembly of the data, data analysis and interpretation, manuscript writing, and final approval of the manuscript. All authors contributed to the article and approved the submitted version.

## Conflict of Interest

The authors declare that the research was conducted in the absence of any commercial or financial relationships that could be construed as a potential conflict of interest.

## Publisher’s Note

All claims expressed in this article are solely those of the authors and do not necessarily represent those of their affiliated organizations, or those of the publisher, the editors and the reviewers. Any product that may be evaluated in this article, or claim that may be made by its manufacturer, is not guaranteed or endorsed by the publisher.

## Footnotes

1
https://www.ncbi.nlm.nih.gov/geo/query/acc.cgi?acc=GSE131411

2
https://commonfund.nih.gov/GTEx/

3
https://www.immport.org/home

4
https://www.gsea-msigdb.org/gsea/msigdb/index.jsp

5
http://cistrome.org/

6
https://github.com/tomastokar/gsoap

7
https://portals.broadinstitute.org/cmap/

8
http://clue.io/

9
https://www.ncbi.nlm.nih.gov/geo/query/acc.cgi?acc=GSE139099

10
https://www.10xgenomics.com/instruments/chromium-x-series

11
http://10xgenomics.com/

12
http://biocc.hrbmu.edu.cn/CellMarker/

13
https://github.com/Coolgenome/iTALK

14
www.r-project.org
