# Supplementary material for: Identification of Key Immune-Related Genes in the Progression of Septic Shock
Source: Front Genet. 2021 Nov 3;12:668527. doi: 10.3389/fgene.2021.668527 (PMC8595268; doi:10.3389/fgene.2021.668527)
Supplement: Supplementary file 1 [file Data_Sheet_1.docx]

setwd("J:\\免疫相关lncRNA\\脓毒症修回")

#########差异分析

setwd("J:\\免疫相关lncRNA\\脓毒症修回\\T1")

rt1=read.table("symbol2.txt",sep="\t",header=T,check.names=F)

rt1 <- as.matrix(rt1)

rownames(rt1)=rt1[,1]

exp=rt1[,2:ncol(rt1)]

dimnames=list(rownames(exp),colnames(exp))

data=matrix(as.numeric(as.matrix(exp)),nrow=nrow(exp),dimnames=dimnames)

data=2^(data)-1

############DEG

data=avereps(data)

data=data[rowMeans(data)>1,]

foldChange=1

padj=0.05

nonmetastasis = 337

metastasis = 21

rt=data #改成自己的文件名

#group=c("normal","tumor","tumor","normal","tumor")

group=c(rep("Non-metastasis",nonmetastasis),rep("Metastasis",metastasis)) #按照癌症和正常样品数目修改

design <- model.matrix(~group)

y <- DGEList(counts=rt,group=group)

y <- calcNormFactors(y)

y <- estimateCommonDisp(y)

y <- estimateTagwiseDisp(y)

et <- exactTest(y,pair = c("Non-metastasis","Metastasis"))

topTags(et)

ordered_tags <- topTags(et, n=100000)

allDiff=ordered_tags$table

allDiff=allDiff[is.na(allDiff$FDR)==FALSE,]

diff=allDiff

newData=y$pseudo.counts

write.table(diff,file="edgerOut.xls",sep="\t",quote=F)

diffSig = diff[(diff$FDR < padj & (diff$logFC>foldChange | diff$logFC<(-foldChange))),]

write.table(diffSig, file="diffSig.xls",sep="\t",quote=F)

diffUp = diff[(diff$FDR < padj & (diff$logFC>foldChange)),]

write.table(diffUp, file="up.xls",sep="\t",quote=F)

diffDown = diff[(diff$FDR < padj & (diff$logFC<(-foldChange))),]

write.table(diffDown, file="down.xls",sep="\t",quote=F)

normalizeExp=rbind(id=colnames(newData),newData)

write.table(normalizeExp,file="normalizeExp.txt",sep="\t",quote=F,col.names=F) #输出所有基因校正后的表达值（normalizeExp.txt）

heatmapData <- newData[rownames(diffSig),]

write.table(heatmapData,file="diffmRNAExp.txt",sep="\t",quote=F)

###################

#colnames(allDiff)[5]= "FDR"

colnames(allDiff)[5]= "FDR"

allDiff$FDR[allDiff$FDR<=0]=2.22329540628561e-322

#volcano

#plot_mode <- "classic" #经典版

plot_mode <- "advanced" #酷炫版

logFCcut <- 0.58 #log2-foldchange

pvalCut <- 0.05 #P.value

adjPcut <- 0.05 #adj.P.value

#for advanced mode

logFCcut2 <- 1

logFCcut3 <- 1.5

pvalCut2 <- 0.00000000000000000001

pvalCut3 <- 0.000000000000000000000000000000000001

#置x，y軸的最大最小位置

xmin <- (range(allDiff$logFC)[1]- (range(allDiff$logFC)[1]+ 20))

xmax <- (range(allDiff$logFC)[1]+ (20-range(allDiff$logFC)[1]))

ymin <- 0

ymax <- 330

# 基因名的颜色，需大于等于pathway的数量，这里自定义了足够多的颜色

mycol <- c("darkgreen","chocolate4","blueviolet","#223D6C","#D20A13","#088247","#58CDD9","#7A142C","#5D90BA","#431A3D","#91612D","#6E568C","#E0367A","#D8D155","#64495D","#7CC767")

###########plot

if (plot_mode == "classic"){

# 簡單的setting for color

allDiff$color_transparent <- ifelse((allDiff$FDR < pvalCut & allDiff$logFC > logFCcut), "red", ifelse((allDiff$FDR < pvalCut & allDiff$logFC < -logFCcut), "blue","grey"))

# 簡單的setting for size

size <- ifelse((allDiff$FDR < pvalCut & abs(allDiff$logFC) > logFCcut), 4, 2)

} else if (plot_mode == "advanced") {

# 複雜的的setting for color

n1 <- length(allDiff[, 1])

cols <- rep("grey", n1)

names(cols)<- rownames(allDiff)

#不同阈值的点的颜色

cols[allDiff$FDR < pvalCut & allDiff$logFC >logFCcut]<- "#FB9A99"

cols[allDiff$FDR < pvalCut2 & allDiff$logFC > logFCcut2]<- "#ED4F4F"

cols[allDiff$FDR < pvalCut & allDiff$logFC < -logFCcut]<- "#B2DF8A"

cols[allDiff$FDR < pvalCut2 & allDiff$logFC < -logFCcut2]<- "#329E3F"

color_transparent <- adjustcolor(cols, alpha.f = 0.5)

allDiff$color_transparent <- color_transparent

# 複雜的的setting for size

n1 <- length(allDiff[, 1])

size <- rep(1, n1)

#不同阈值的点的大小

size[allDiff$FDR < pvalCut & allDiff$logFC > logFCcut]<- 1

size[allDiff$FDR < pvalCut2 & allDiff$logFC > logFCcut2]<- 2

size[allDiff$FDR < pvalCut3 & allDiff$logFC > logFCcut3]<- 3

size[allDiff$FDR < pvalCut & allDiff$logFC < -logFCcut]<- 1

size[allDiff$FDR < pvalCut2 & allDiff$logFC < -logFCcut2]<- 2

size[allDiff$FDR < pvalCut3 & allDiff$logFC < -logFCcut3]<- 3

} else {

stop("Unsupport mode")

}

# Construct the plot object

p1 <- ggplot(data=allDiff, aes(logFC, -log10(FDR))) +

geom_point(alpha = 0.6, size = size, colour = allDiff$color_transparent) +

labs(allDiff=bquote(~Log[2]~"(fold change)"), y=bquote(~-Log[10]~italic("P-value")), title="") +

ylim(c(ymin,ymax)) +

scale_x_continuous(

breaks = c(-10, -5, -logFCcut, 0, logFCcut, 5, 10), #刻度线的位置

labels = c(-10, -5, -logFCcut, 0, logFCcut, 5, 10),

limits = c(-11, 11) #x轴范围，两侧对称才好看

) +

#或用下面这行：

xlim(c(xmin, xmax)) +

#画阈值分界线

geom_vline(xintercept = c(-logFCcut, logFCcut), color="grey40",

linetype="longdash", lwd = 0.5) + #虚线的形状和粗细

geom_hline(yintercept = -log10(pvalCut), color="grey40",

linetype="longdash", lwd = 0.5) +

theme_bw(base_size = 12#, base_family = "Times" #修改字体

) +

theme(panel.grid=element_blank())

if (plot_mode == "advanced") {

p1 <- p1 +

geom_vline(xintercept = c(-logFCcut2, logFCcut2), color="grey40",

linetype="longdash", lwd = 0.5) +

geom_hline(yintercept = -log10(pvalCut2), color="grey40",

linetype="longdash", lwd = 0.5)

}

pdf(file="vol.pdf",5,5)

p1

dev.off()

#heatmap

fdrFilter=0.05

logFCfilter=1

nonmetastasis = 337

metastasis = 21

rt=diffSig

diffExp=read.table("Group_symbol_FPKM1.txt",sep="\t",header=T,check.names=F)

diffExp <- as.matrix(diffExp)

rownames(diffExp)=diffExp[,1]

exp=diffExp[,2:ncol(diffExp)]

dimnames=list(rownames(exp),colnames(exp))

diffExp=matrix(as.numeric(as.matrix(exp)),nrow=nrow(exp),dimnames=dimnames)

gene=rownames(diffSig)

immuneDiffAll=rt[intersect(gene,rownames(rt)),]

immuneDiffGene=intersect(gene,rownames(diffSig))

hmExp=newData[immuneDiffGene,]

immuneDiffResult=immuneDiffAll[immuneDiffGene,]

immuneDiffResult=rbind(ID=colnames(immuneDiffResult),immuneDiffResult)

write.table(immuneDiffResult,file="Key_Diff_DEG.xls",sep="\t",col.names=F,quote=F)

############

Type=read.table("clinical.txt",sep="\t",header=T,check.names=F,row.names = 1)

Type=as.data.frame(Type)

hmExp=heatmapData

hmExp=log2(hmExp+1)

plotdata = t(hmExp)

# rainbow是R的一个函数，用于产生彩虹色

E = normalizeBetweenArrays(plotdata, method="quantile")

plotdata = E

pdf(file="heatmap_keygene.pdf",height=7.5,width=10)

pheatmap(t(plotdata),

annotation=Type,

color = colorRampPalette(c("#3F007D", "white", "#E34A33"))(50),

cluster_cols =F,

cluster_rows = T,

show_colnames = F,

show_rownames = F,

fontsize = 12,

fontsize_row=8,

fontsize_col=10)

dev.off()

#############

library(clusterProfiler)

library(msigdbr)

library(DOSE)

library(enrichplot)

library(ggplot2)

library(plyr)

library(dplyr)

gsym.fc = diff

gsym.fc[1:3,]

#rownames(gsym.fc) = gsym.fc$id

# cutoff

logFCcut <- 1 #log2-foldchange

adjPcut <- 0.05 #adj.P.value

# 筛选差异基因

geneup <- rownames(gsym.fc[gsym.fc$logFC > logFCcut & gsym.fc$FDR < adjPcut, ])

genedown <- rownames(gsym.fc[gsym.fc$logFC < -logFCcut & gsym.fc$FDR < adjPcut, ])

# 数量

length(geneup)

# 保存到文件，便于套用格式

write.table(geneup, "easy_input_up.txt", quote = F, row.names = F, col.names = F)

write.table(genedown, "easy_input_down.txt", quote = F, row.names = F, col.names = F)

#ORA富集分析

# 自定义函数

enrich_func <- function(x, gene, readable = FALSE) {

en_result <- enricher(gene, TERM2GENE = x)

if (readable & nrow(en_result) > 0)

en_result <- setReadable(en_result, 'org.Hs.eg.db', #物种

'ENTREZID')

return(en_result)

}

## 数据处理函数

merge_result2 <- function(enrichResultList, output = "compareClusterResult") {

if ( !is(enrichResultList, "list")) {

stop("input should be a name list...")

}

if ( is.null(names(enrichResultList))) {

stop("input should be a name list...")

}

x <- lapply(enrichResultList, as.data.frame)

names(x) <- names(enrichResultList)

y <- ldply(x, "rbind")

if (output == "compareClusterResult") {

y <- plyr::rename(y, c(.id="Cluster"))

y$Cluster = factor(y$Cluster, levels=names(enrichResultList))

return(new("compareClusterResult",

compareClusterResult = y))

}

y <- plyr::rename(y, c(.id="Category"))

if (output == "enrichResult") {

return(new("enrichResult",

result = y))

}

if (output == "gseaResult") {

return(new("gseaResult",

result = y))

}

}

keep_category <- function(em_ORA, n) {

table_em <- as.numeric(table(em_ORA$Category))

start <- rep(0, length(table_em) - 1)

for(i in seq_len(length(table_em) - 1)) {

start[i] <- sum(table_em[seq_len(i)])

}

showCategorys <- sapply(table_em, function(x) min(n, x))

start <- c(0, start) + 1

end <- start + showCategorys - 1

keep <- NULL

for(i in seq_len(length(start))) {

keep <- c(keep, c(start[i] : end[i]))

}

return(keep)

}

enrich_filter <- function(em_result, showCategory) {

keep <- keep_category(em_result, showCategory)

em_result <- em_result[keep, ]

if ("NES" %in% colnames(em_result))

em_result$Count <- em_result$core_enrichment %>%

strsplit(split = "/") %>%

vapply(length, FUN.VALUE = 1)

return(em_result)

}

## 作图函数

em_plot <- function(em_1 = NULL, em_2 = NULL, showCategory = 2, fill = "p.adjust", hjust = 1) {

fill <- match.arg(fill, c("Category", "p.adjust", "log10_p.adjust"))

result1 <- enrich_filter(em_1, showCategory)

if (is.null(em_2)) {

result <- result1

} else {

result2 <- enrich_filter(em_2, showCategory)

result2$Count <- -result2$Count

result <- rbind(result1, result2)

}

result$Category <- gsub("\n.*", "", result$Category)

result$log10_p.adjust <- log10(result$p.adjust)

data_plot <- result[, c("ID", "Category", "p.adjust", "log10_p.adjust", "Count")]

data_plot2 <- data_plot

data_plot2$ID <- factor(data_plot2$ID, levels = unique(data_plot2$ID))

data_plot2 <- plyr::rename(data_plot2, c("Count" = "gene_number"))

h_just <- ifelse(data_plot2$gene_number < 0, -hjust, hjust)

ggplot(data_plot2, aes_string(x = "gene_number", y = "ID", fill = fill)) +

geom_col() +

geom_text(aes_(x =~ gene_number + h_just, label =~ abs(gene_number)),

color="black") +

scale_x_continuous(label = abs,

expand = expansion(mult = c(.01, .01))) + #两侧留空

theme_classic() +

ylab("") +

theme(axis.title.x = element_text(size = 15)) +

facet_grid(Category ~ ., scales="free", space="free")

}

# 以人为例

#msigdbr_species()

gmt <- msigdbr(species = "Homo sapiens")

gmt2 <- gmt%>%

dplyr::select(gs_name, entrez_gene)

gmts <- split(gmt2, gmt$gs_cat)

# 先以上调表达的基因为例

# 加载基因名

geneup <- read.table("easy_input_up.txt")$V1

# 把gene symbol转换为ENTREZ ID

# 此处物种是人，其他物种的ID转换方法，请参考FigureYa52GOplot

geneup.id <- bitr(geneup, fromType = "SYMBOL", toType = "ENTREZID", OrgDb = "org.Hs.eg.db")

# 富集分析

em_ORAup <- setNames(lapply(gmts, enrich_func, geneup.id$ENTREZID, readable = TRUE), names(gmts)) %>%

merge_result2(output = "enrichResult")

# 输出到文件

write.csv(em_ORAup@result, "output_ORA_up.csv", quote = F)

#下调表达的基因也是一样的操作

genedown <- read.table("easy_input_down.txt")$V1

genedown.id <- bitr(genedown, fromType = "SYMBOL", toType = "ENTREZID", OrgDb = "org.Hs.eg.db")

em_ORAdown <- setNames(lapply(gmts, enrich_func, genedown.id$ENTREZID, readable = TRUE), names(gmts)) %>%

merge_result2(output = "enrichResult")

# 上调表达基因的富集结果

pdf(file="ORA_up_DEG.pdf",width = 10,height = 8)

em_plot(em_ORAup, showCategory = 3, fill = "Category", hjust = 10) + scale_fill_brewer(palette="Set1")

dev.off()

# 下调表达基因的富集结果

pdf(file="ORA_down_DEG.pdf",width = 10,height = 8)

em_plot(em_ORAdown, showCategory = 3, fill = "Category", hjust = 5) + scale_fill_brewer(palette="Set1")

dev.off()

# 上调的画在右侧，下调的画在左侧

pdf(file="ORA_ALL_DEG.pdf",width = 10,height = 8)

em_plot(em_ORAup, em_ORAdown, showCategory = 3, fill = "Category", hjust = 12) + scale_fill_brewer(palette="Set1")

dev.off()

# 每种注释筛选2个term

keep1 <- keep_category(em_ORAup, 2)

keep2 <- keep_category(em_ORAdown, 2)

em_ora1 <- em_ORAup[keep1, ]

em_ora2 <- em_ORAdown[keep2, ]

# 上调画右边，下调画左边

em_ora2$Count <- -em_ora2$Count

em_ora <- new("enrichResult",

result = rbind(em_ora1, em_ora2))

# 画barplot

#barplot(em_ora, showCategory = nrow(em_ora)) +

# scale_x_continuous(label = abs) +

# facet_grid(Category ~ ., scales="free",space="free")

# 画dotplot

pdf(file="ORA_ALL_DEG_doplot.pdf",width = 12,height = 8)

dotplot(em_ora, showCategory = nrow(em_ora)) +

scale_x_continuous(label = abs) +

facet_grid(Category ~ ., scales="free",space="free")

dev.off()

#########差异分析

setwd("J:\\免疫相关lncRNA\\脓毒症修回\\T2")

rt1=read.table("symbol2.txt",sep="\t",header=T,check.names=F)

rt1 <- as.matrix(rt1)

rownames(rt1)=rt1[,1]

exp=rt1[,2:ncol(rt1)]

dimnames=list(rownames(exp),colnames(exp))

data=matrix(as.numeric(as.matrix(exp)),nrow=nrow(exp),dimnames=dimnames)

data=2^(data)-1

############DEG

data=avereps(data)

data=data[rowMeans(data)>1,]

foldChange=1

padj=0.05

nonmetastasis = 337

metastasis = 21

rt=data #改成自己的文件名

#group=c("normal","tumor","tumor","normal","tumor")

group=c(rep("Non-metastasis",nonmetastasis),rep("Metastasis",metastasis)) #按照癌症和正常样品数目修改

design <- model.matrix(~group)

y <- DGEList(counts=rt,group=group)

y <- calcNormFactors(y)

y <- estimateCommonDisp(y)

y <- estimateTagwiseDisp(y)

et <- exactTest(y,pair = c("Non-metastasis","Metastasis"))

topTags(et)

ordered_tags <- topTags(et, n=100000)

allDiff=ordered_tags$table

allDiff=allDiff[is.na(allDiff$FDR)==FALSE,]

diff=allDiff

newData=y$pseudo.counts

write.table(diff,file="edgerOut.xls",sep="\t",quote=F)

diffSig = diff[(diff$FDR < padj & (diff$logFC>foldChange | diff$logFC<(-foldChange))),]

write.table(diffSig, file="diffSig.xls",sep="\t",quote=F)

diffUp = diff[(diff$FDR < padj & (diff$logFC>foldChange)),]

write.table(diffUp, file="up.xls",sep="\t",quote=F)

diffDown = diff[(diff$FDR < padj & (diff$logFC<(-foldChange))),]

write.table(diffDown, file="down.xls",sep="\t",quote=F)

normalizeExp=rbind(id=colnames(newData),newData)

write.table(normalizeExp,file="normalizeExp.txt",sep="\t",quote=F,col.names=F) #输出所有基因校正后的表达值（normalizeExp.txt）

heatmapData <- newData[rownames(diffSig),]

write.table(heatmapData,file="diffmRNAExp.txt",sep="\t",quote=F)

###################

#colnames(allDiff)[5]= "FDR"

colnames(allDiff)[5]= "FDR"

allDiff$FDR[allDiff$FDR<=0]=2.22329540628561e-322

#volcano

#plot_mode <- "classic" #经典版

plot_mode <- "advanced" #酷炫版

logFCcut <- 0.58 #log2-foldchange

pvalCut <- 0.05 #P.value

adjPcut <- 0.05 #adj.P.value

#for advanced mode

logFCcut2 <- 1

logFCcut3 <- 1.5

pvalCut2 <- 0.00000000000000000001

pvalCut3 <- 0.000000000000000000000000000000000001

#置x，y軸的最大最小位置

xmin <- (range(allDiff$logFC)[1]- (range(allDiff$logFC)[1]+ 20))

xmax <- (range(allDiff$logFC)[1]+ (20-range(allDiff$logFC)[1]))

ymin <- 0

ymax <- 330

# 基因名的颜色，需大于等于pathway的数量，这里自定义了足够多的颜色

mycol <- c("darkgreen","chocolate4","blueviolet","#223D6C","#D20A13","#088247","#58CDD9","#7A142C","#5D90BA","#431A3D","#91612D","#6E568C","#E0367A","#D8D155","#64495D","#7CC767")

###########plot

if (plot_mode == "classic"){

# 簡單的setting for color

allDiff$color_transparent <- ifelse((allDiff$FDR < pvalCut & allDiff$logFC > logFCcut), "red", ifelse((allDiff$FDR < pvalCut & allDiff$logFC < -logFCcut), "blue","grey"))

# 簡單的setting for size

size <- ifelse((allDiff$FDR < pvalCut & abs(allDiff$logFC) > logFCcut), 4, 2)

} else if (plot_mode == "advanced") {

# 複雜的的setting for color

n1 <- length(allDiff[, 1])

cols <- rep("grey", n1)

names(cols)<- rownames(allDiff)

#不同阈值的点的颜色

cols[allDiff$FDR < pvalCut & allDiff$logFC >logFCcut]<- "#FB9A99"

cols[allDiff$FDR < pvalCut2 & allDiff$logFC > logFCcut2]<- "#ED4F4F"

cols[allDiff$FDR < pvalCut & allDiff$logFC < -logFCcut]<- "#B2DF8A"

cols[allDiff$FDR < pvalCut2 & allDiff$logFC < -logFCcut2]<- "#329E3F"

color_transparent <- adjustcolor(cols, alpha.f = 0.5)

allDiff$color_transparent <- color_transparent

# 複雜的的setting for size

n1 <- length(allDiff[, 1])

size <- rep(1, n1)

#不同阈值的点的大小

size[allDiff$FDR < pvalCut & allDiff$logFC > logFCcut]<- 1

size[allDiff$FDR < pvalCut2 & allDiff$logFC > logFCcut2]<- 2

size[allDiff$FDR < pvalCut3 & allDiff$logFC > logFCcut3]<- 3

size[allDiff$FDR < pvalCut & allDiff$logFC < -logFCcut]<- 1

size[allDiff$FDR < pvalCut2 & allDiff$logFC < -logFCcut2]<- 2

size[allDiff$FDR < pvalCut3 & allDiff$logFC < -logFCcut3]<- 3

} else {

stop("Unsupport mode")

}

# Construct the plot object

p1 <- ggplot(data=allDiff, aes(logFC, -log10(FDR))) +

geom_point(alpha = 0.6, size = size, colour = allDiff$color_transparent) +

labs(allDiff=bquote(~Log[2]~"(fold change)"), y=bquote(~-Log[10]~italic("P-value")), title="") +

ylim(c(ymin,ymax)) +

scale_x_continuous(

breaks = c(-10, -5, -logFCcut, 0, logFCcut, 5, 10), #刻度线的位置

labels = c(-10, -5, -logFCcut, 0, logFCcut, 5, 10),

limits = c(-11, 11) #x轴范围，两侧对称才好看

) +

#或用下面这行：

xlim(c(xmin, xmax)) +

#画阈值分界线

geom_vline(xintercept = c(-logFCcut, logFCcut), color="grey40",

linetype="longdash", lwd = 0.5) + #虚线的形状和粗细

geom_hline(yintercept = -log10(pvalCut), color="grey40",

linetype="longdash", lwd = 0.5) +

theme_bw(base_size = 12#, base_family = "Times" #修改字体

) +

theme(panel.grid=element_blank())

if (plot_mode == "advanced") {

p1 <- p1 +

geom_vline(xintercept = c(-logFCcut2, logFCcut2), color="grey40",

linetype="longdash", lwd = 0.5) +

geom_hline(yintercept = -log10(pvalCut2), color="grey40",

linetype="longdash", lwd = 0.5)

}

pdf(file="vol.pdf",5,5)

p1

dev.off()

############

Type=read.table("clinical.txt",sep="\t",header=T,check.names=F,row.names = 1)

Type=as.data.frame(Type)

hmExp=heatmapData

hmExp=log2(hmExp+1)

plotdata = t(hmExp)

# rainbow是R的一个函数，用于产生彩虹色

E = normalizeBetweenArrays(plotdata, method="quantile")

plotdata = E

pdf(file="heatmap_keygene.pdf",height=7.5,width=10)

pheatmap(t(plotdata),

annotation=Type,

color = colorRampPalette(c("#3F007D", "white", "#E34A33"))(50),

cluster_cols =F,

cluster_rows = T,

show_colnames = F,

show_rownames = F,

fontsize = 12,

fontsize_row=8,

fontsize_col=10)

dev.off()

#############

library(clusterProfiler)

library(msigdbr)

library(DOSE)

library(enrichplot)

library(ggplot2)

library(plyr)

library(dplyr)

gsym.fc = diff

gsym.fc[1:3,]

#rownames(gsym.fc) = gsym.fc$id

# cutoff

logFCcut <- 1 #log2-foldchange

adjPcut <- 0.05 #adj.P.value

# 筛选差异基因

geneup <- rownames(gsym.fc[gsym.fc$logFC > logFCcut & gsym.fc$FDR < adjPcut, ])

genedown <- rownames(gsym.fc[gsym.fc$logFC < -logFCcut & gsym.fc$FDR < adjPcut, ])

# 数量

length(geneup)

# 保存到文件，便于套用格式

write.table(geneup, "easy_input_up.txt", quote = F, row.names = F, col.names = F)

write.table(genedown, "easy_input_down.txt", quote = F, row.names = F, col.names = F)

#ORA富集分析

# 自定义函数

enrich_func <- function(x, gene, readable = FALSE) {

en_result <- enricher(gene, TERM2GENE = x)

if (readable & nrow(en_result) > 0)

en_result <- setReadable(en_result, 'org.Hs.eg.db', #物种

'ENTREZID')

return(en_result)

}

## 数据处理函数

merge_result2 <- function(enrichResultList, output = "compareClusterResult") {

if ( !is(enrichResultList, "list")) {

stop("input should be a name list...")

}

if ( is.null(names(enrichResultList))) {

stop("input should be a name list...")

}

x <- lapply(enrichResultList, as.data.frame)

names(x) <- names(enrichResultList)

y <- ldply(x, "rbind")

if (output == "compareClusterResult") {

y <- plyr::rename(y, c(.id="Cluster"))

y$Cluster = factor(y$Cluster, levels=names(enrichResultList))

return(new("compareClusterResult",

compareClusterResult = y))

}

y <- plyr::rename(y, c(.id="Category"))

if (output == "enrichResult") {

return(new("enrichResult",

result = y))

}

if (output == "gseaResult") {

return(new("gseaResult",

result = y))

}

}

keep_category <- function(em_ORA, n) {

table_em <- as.numeric(table(em_ORA$Category))

start <- rep(0, length(table_em) - 1)

for(i in seq_len(length(table_em) - 1)) {

start[i] <- sum(table_em[seq_len(i)])

}

showCategorys <- sapply(table_em, function(x) min(n, x))

start <- c(0, start) + 1

end <- start + showCategorys - 1

keep <- NULL

for(i in seq_len(length(start))) {

keep <- c(keep, c(start[i] : end[i]))

}

return(keep)

}

enrich_filter <- function(em_result, showCategory) {

keep <- keep_category(em_result, showCategory)

em_result <- em_result[keep, ]

if ("NES" %in% colnames(em_result))

em_result$Count <- em_result$core_enrichment %>%

strsplit(split = "/") %>%

vapply(length, FUN.VALUE = 1)

return(em_result)

}

## 作图函数

em_plot <- function(em_1 = NULL, em_2 = NULL, showCategory = 2, fill = "p.adjust", hjust = 1) {

fill <- match.arg(fill, c("Category", "p.adjust", "log10_p.adjust"))

result1 <- enrich_filter(em_1, showCategory)

if (is.null(em_2)) {

result <- result1

} else {

result2 <- enrich_filter(em_2, showCategory)

result2$Count <- -result2$Count

result <- rbind(result1, result2)

}

result$Category <- gsub("\n.*", "", result$Category)

result$log10_p.adjust <- log10(result$p.adjust)

data_plot <- result[, c("ID", "Category", "p.adjust", "log10_p.adjust", "Count")]

data_plot2 <- data_plot

data_plot2$ID <- factor(data_plot2$ID, levels = unique(data_plot2$ID))

data_plot2 <- plyr::rename(data_plot2, c("Count" = "gene_number"))

h_just <- ifelse(data_plot2$gene_number < 0, -hjust, hjust)

ggplot(data_plot2, aes_string(x = "gene_number", y = "ID", fill = fill)) +

geom_col() +

geom_text(aes_(x =~ gene_number + h_just, label =~ abs(gene_number)),

color="black") +

scale_x_continuous(label = abs,

expand = expansion(mult = c(.01, .01))) + #两侧留空

theme_classic() +

ylab("") +

theme(axis.title.x = element_text(size = 15)) +

facet_grid(Category ~ ., scales="free", space="free")

}

# 以人为例

#msigdbr_species()

gmt <- msigdbr(species = "Homo sapiens")

gmt2 <- gmt%>%

dplyr::select(gs_name, entrez_gene)

gmts <- split(gmt2, gmt$gs_cat)

# 先以上调表达的基因为例

# 加载基因名

geneup <- read.table("easy_input_up.txt")$V1

# 把gene symbol转换为ENTREZ ID

# 此处物种是人，其他物种的ID转换方法，请参考FigureYa52GOplot

geneup.id <- bitr(geneup, fromType = "SYMBOL", toType = "ENTREZID", OrgDb = "org.Hs.eg.db")

# 富集分析

em_ORAup <- setNames(lapply(gmts, enrich_func, geneup.id$ENTREZID, readable = TRUE), names(gmts)) %>%

merge_result2(output = "enrichResult")

# 输出到文件

write.csv(em_ORAup@result, "output_ORA_up.csv", quote = F)

#下调表达的基因也是一样的操作

genedown <- read.table("easy_input_down.txt")$V1

genedown.id <- bitr(genedown, fromType = "SYMBOL", toType = "ENTREZID", OrgDb = "org.Hs.eg.db")

em_ORAdown <- setNames(lapply(gmts, enrich_func, genedown.id$ENTREZID, readable = TRUE), names(gmts)) %>%

merge_result2(output = "enrichResult")

# 上调表达基因的富集结果

pdf(file="ORA_up_DEG.pdf",width = 10,height = 8)

em_plot(em_ORAup, showCategory = 3, fill = "Category", hjust = 10) + scale_fill_brewer(palette="Set1")

dev.off()

# 下调表达基因的富集结果

pdf(file="ORA_down_DEG.pdf",width = 10,height = 8)

em_plot(em_ORAdown, showCategory = 3, fill = "Category", hjust = 5) + scale_fill_brewer(palette="Set1")

dev.off()

# 上调的画在右侧，下调的画在左侧

pdf(file="ORA_ALL_DEG.pdf",width = 10,height = 8)

em_plot(em_ORAup, em_ORAdown, showCategory = 3, fill = "Category", hjust = 12) + scale_fill_brewer(palette="Set1")

dev.off()

# 每种注释筛选2个term

keep1 <- keep_category(em_ORAup, 2)

keep2 <- keep_category(em_ORAdown, 2)

em_ora1 <- em_ORAup[keep1, ]

em_ora2 <- em_ORAdown[keep2, ]

# 上调画右边，下调画左边

em_ora2$Count <- -em_ora2$Count

em_ora <- new("enrichResult",

result = rbind(em_ora1, em_ora2))

# 画barplot

#barplot(em_ora, showCategory = nrow(em_ora)) +

# scale_x_continuous(label = abs) +

# facet_grid(Category ~ ., scales="free",space="free")

# 画dotplot

pdf(file="ORA_ALL_DEG_doplot.pdf",width = 12,height = 8)

dotplot(em_ora, showCategory = nrow(em_ora)) +

scale_x_continuous(label = abs) +

facet_grid(Category ~ ., scales="free",space="free")

dev.off()

#########差异分析

setwd("J:\\免疫相关lncRNA\\脓毒症修回\\T3")

rt1=read.table("symbol2.txt",sep="\t",header=T,check.names=F)

rt1 <- as.matrix(rt1)

rownames(rt1)=rt1[,1]

exp=rt1[,2:ncol(rt1)]

dimnames=list(rownames(exp),colnames(exp))

data=matrix(as.numeric(as.matrix(exp)),nrow=nrow(exp),dimnames=dimnames)

data=2^(data)-1

############DEG

data=avereps(data)

data=data[rowMeans(data)>1,]

foldChange=1

padj=0.05

nonmetastasis = 337

metastasis = 21

rt=data #改成自己的文件名

#group=c("normal","tumor","tumor","normal","tumor")

group=c(rep("Non-metastasis",nonmetastasis),rep("Metastasis",metastasis)) #按照癌症和正常样品数目修改

design <- model.matrix(~group)

y <- DGEList(counts=rt,group=group)

y <- calcNormFactors(y)

y <- estimateCommonDisp(y)

y <- estimateTagwiseDisp(y)

et <- exactTest(y,pair = c("Non-metastasis","Metastasis"))

topTags(et)

ordered_tags <- topTags(et, n=100000)

allDiff=ordered_tags$table

allDiff=allDiff[is.na(allDiff$FDR)==FALSE,]

diff=allDiff

newData=y$pseudo.counts

write.table(diff,file="edgerOut.xls",sep="\t",quote=F)

diffSig = diff[(diff$FDR < padj & (diff$logFC>foldChange | diff$logFC<(-foldChange))),]

write.table(diffSig, file="diffSig.xls",sep="\t",quote=F)

diffUp = diff[(diff$FDR < padj & (diff$logFC>foldChange)),]

write.table(diffUp, file="up.xls",sep="\t",quote=F)

diffDown = diff[(diff$FDR < padj & (diff$logFC<(-foldChange))),]

write.table(diffDown, file="down.xls",sep="\t",quote=F)

normalizeExp=rbind(id=colnames(newData),newData)

write.table(normalizeExp,file="normalizeExp.txt",sep="\t",quote=F,col.names=F) #输出所有基因校正后的表达值（normalizeExp.txt）

heatmapData <- newData[rownames(diffSig),]

write.table(heatmapData,file="diffmRNAExp.txt",sep="\t",quote=F)

###################

#colnames(allDiff)[5]= "FDR"

colnames(allDiff)[5]= "FDR"

allDiff$FDR[allDiff$FDR<=0]=2.22329540628561e-322

#volcano

#plot_mode <- "classic" #经典版

plot_mode <- "advanced" #酷炫版

logFCcut <- 0.58 #log2-foldchange

pvalCut <- 0.05 #P.value

adjPcut <- 0.05 #adj.P.value

#for advanced mode

logFCcut2 <- 1

logFCcut3 <- 1.5

pvalCut2 <- 0.00000000000000000001

pvalCut3 <- 0.000000000000000000000000000000000001

#置x，y軸的最大最小位置

xmin <- (range(allDiff$logFC)[1]- (range(allDiff$logFC)[1]+ 20))

xmax <- (range(allDiff$logFC)[1]+ (20-range(allDiff$logFC)[1]))

ymin <- 0

ymax <- 330

# 基因名的颜色，需大于等于pathway的数量，这里自定义了足够多的颜色

mycol <- c("darkgreen","chocolate4","blueviolet","#223D6C","#D20A13","#088247","#58CDD9","#7A142C","#5D90BA","#431A3D","#91612D","#6E568C","#E0367A","#D8D155","#64495D","#7CC767")

###########plot

if (plot_mode == "classic"){

# 簡單的setting for color

allDiff$color_transparent <- ifelse((allDiff$FDR < pvalCut & allDiff$logFC > logFCcut), "red", ifelse((allDiff$FDR < pvalCut & allDiff$logFC < -logFCcut), "blue","grey"))

# 簡單的setting for size

size <- ifelse((allDiff$FDR < pvalCut & abs(allDiff$logFC) > logFCcut), 4, 2)

} else if (plot_mode == "advanced") {

# 複雜的的setting for color

n1 <- length(allDiff[, 1])

cols <- rep("grey", n1)

names(cols)<- rownames(allDiff)

#不同阈值的点的颜色

cols[allDiff$FDR < pvalCut & allDiff$logFC >logFCcut]<- "#FB9A99"

cols[allDiff$FDR < pvalCut2 & allDiff$logFC > logFCcut2]<- "#ED4F4F"

cols[allDiff$FDR < pvalCut & allDiff$logFC < -logFCcut]<- "#B2DF8A"

cols[allDiff$FDR < pvalCut2 & allDiff$logFC < -logFCcut2]<- "#329E3F"

color_transparent <- adjustcolor(cols, alpha.f = 0.5)

allDiff$color_transparent <- color_transparent

# 複雜的的setting for size

n1 <- length(allDiff[, 1])

size <- rep(1, n1)

#不同阈值的点的大小

size[allDiff$FDR < pvalCut & allDiff$logFC > logFCcut]<- 1

size[allDiff$FDR < pvalCut2 & allDiff$logFC > logFCcut2]<- 2

size[allDiff$FDR < pvalCut3 & allDiff$logFC > logFCcut3]<- 3

size[allDiff$FDR < pvalCut & allDiff$logFC < -logFCcut]<- 1

size[allDiff$FDR < pvalCut2 & allDiff$logFC < -logFCcut2]<- 2

size[allDiff$FDR < pvalCut3 & allDiff$logFC < -logFCcut3]<- 3

} else {

stop("Unsupport mode")

}

# Construct the plot object

p1 <- ggplot(data=allDiff, aes(logFC, -log10(FDR))) +

geom_point(alpha = 0.6, size = size, colour = allDiff$color_transparent) +

labs(allDiff=bquote(~Log[2]~"(fold change)"), y=bquote(~-Log[10]~italic("P-value")), title="") +

ylim(c(ymin,ymax)) +

scale_x_continuous(

breaks = c(-10, -5, -logFCcut, 0, logFCcut, 5, 10), #刻度线的位置

labels = c(-10, -5, -logFCcut, 0, logFCcut, 5, 10),

limits = c(-11, 11) #x轴范围，两侧对称才好看

) +

#或用下面这行：

xlim(c(xmin, xmax)) +

#画阈值分界线

geom_vline(xintercept = c(-logFCcut, logFCcut), color="grey40",

linetype="longdash", lwd = 0.5) + #虚线的形状和粗细

geom_hline(yintercept = -log10(pvalCut), color="grey40",

linetype="longdash", lwd = 0.5) +

theme_bw(base_size = 12#, base_family = "Times" #修改字体

) +

theme(panel.grid=element_blank())

if (plot_mode == "advanced") {

p1 <- p1 +

geom_vline(xintercept = c(-logFCcut2, logFCcut2), color="grey40",

linetype="longdash", lwd = 0.5) +

geom_hline(yintercept = -log10(pvalCut2), color="grey40",

linetype="longdash", lwd = 0.5)

}

pdf(file="vol.pdf",5,5)

p1

dev.off()

############

Type=read.table("clinical.txt",sep="\t",header=T,check.names=F,row.names = 1)

Type=as.data.frame(Type)

hmExp=heatmapData

hmExp=log2(hmExp+1)

plotdata = t(hmExp)

# rainbow是R的一个函数，用于产生彩虹色

E = normalizeBetweenArrays(plotdata, method="quantile")

plotdata = E

pdf(file="heatmap_keygene.pdf",height=7.5,width=10)

pheatmap(t(plotdata),

annotation=Type,

color = colorRampPalette(c("#3F007D", "white", "#E34A33"))(50),

cluster_cols =F,

cluster_rows = T,

show_colnames = F,

show_rownames = F,

fontsize = 12,

fontsize_row=8,

fontsize_col=10)

dev.off()

#############

library(clusterProfiler)

library(msigdbr)

library(DOSE)

library(enrichplot)

library(ggplot2)

library(plyr)

library(dplyr)

gsym.fc = diff

gsym.fc[1:3,]

#rownames(gsym.fc) = gsym.fc$id

# cutoff

logFCcut <- 1 #log2-foldchange

adjPcut <- 0.05 #adj.P.value

# 筛选差异基因

geneup <- rownames(gsym.fc[gsym.fc$logFC > logFCcut & gsym.fc$FDR < adjPcut, ])

genedown <- rownames(gsym.fc[gsym.fc$logFC < -logFCcut & gsym.fc$FDR < adjPcut, ])

# 数量

length(geneup)

# 保存到文件，便于套用格式

write.table(geneup, "easy_input_up.txt", quote = F, row.names = F, col.names = F)

write.table(genedown, "easy_input_down.txt", quote = F, row.names = F, col.names = F)

#ORA富集分析

# 自定义函数

enrich_func <- function(x, gene, readable = FALSE) {

en_result <- enricher(gene, TERM2GENE = x)

if (readable & nrow(en_result) > 0)

en_result <- setReadable(en_result, 'org.Hs.eg.db', #物种

'ENTREZID')

return(en_result)

}

## 数据处理函数

merge_result2 <- function(enrichResultList, output = "compareClusterResult") {

if ( !is(enrichResultList, "list")) {

stop("input should be a name list...")

}

if ( is.null(names(enrichResultList))) {

stop("input should be a name list...")

}

x <- lapply(enrichResultList, as.data.frame)

names(x) <- names(enrichResultList)

y <- ldply(x, "rbind")

if (output == "compareClusterResult") {

y <- plyr::rename(y, c(.id="Cluster"))

y$Cluster = factor(y$Cluster, levels=names(enrichResultList))

return(new("compareClusterResult",

compareClusterResult = y))

}

y <- plyr::rename(y, c(.id="Category"))

if (output == "enrichResult") {

return(new("enrichResult",

result = y))

}

if (output == "gseaResult") {

return(new("gseaResult",

result = y))

}

}

keep_category <- function(em_ORA, n) {

table_em <- as.numeric(table(em_ORA$Category))

start <- rep(0, length(table_em) - 1)

for(i in seq_len(length(table_em) - 1)) {

start[i] <- sum(table_em[seq_len(i)])

}

showCategorys <- sapply(table_em, function(x) min(n, x))

start <- c(0, start) + 1

end <- start + showCategorys - 1

keep <- NULL

for(i in seq_len(length(start))) {

keep <- c(keep, c(start[i] : end[i]))

}

return(keep)

}

enrich_filter <- function(em_result, showCategory) {

keep <- keep_category(em_result, showCategory)

em_result <- em_result[keep, ]

if ("NES" %in% colnames(em_result))

em_result$Count <- em_result$core_enrichment %>%

strsplit(split = "/") %>%

vapply(length, FUN.VALUE = 1)

return(em_result)

}

## 作图函数

em_plot <- function(em_1 = NULL, em_2 = NULL, showCategory = 2, fill = "p.adjust", hjust = 1) {

fill <- match.arg(fill, c("Category", "p.adjust", "log10_p.adjust"))

result1 <- enrich_filter(em_1, showCategory)

if (is.null(em_2)) {

result <- result1

} else {

result2 <- enrich_filter(em_2, showCategory)

result2$Count <- -result2$Count

result <- rbind(result1, result2)

}

result$Category <- gsub("\n.*", "", result$Category)

result$log10_p.adjust <- log10(result$p.adjust)

data_plot <- result[, c("ID", "Category", "p.adjust", "log10_p.adjust", "Count")]

data_plot2 <- data_plot

data_plot2$ID <- factor(data_plot2$ID, levels = unique(data_plot2$ID))

data_plot2 <- plyr::rename(data_plot2, c("Count" = "gene_number"))

h_just <- ifelse(data_plot2$gene_number < 0, -hjust, hjust)

ggplot(data_plot2, aes_string(x = "gene_number", y = "ID", fill = fill)) +

geom_col() +

geom_text(aes_(x =~ gene_number + h_just, label =~ abs(gene_number)),

color="black") +

scale_x_continuous(label = abs,

expand = expansion(mult = c(.01, .01))) + #两侧留空

theme_classic() +

ylab("") +

theme(axis.title.x = element_text(size = 15)) +

facet_grid(Category ~ ., scales="free", space="free")

}

# 以人为例

#msigdbr_species()

gmt <- msigdbr(species = "Homo sapiens")

gmt2 <- gmt%>%

dplyr::select(gs_name, entrez_gene)

gmts <- split(gmt2, gmt$gs_cat)

# 先以上调表达的基因为例

# 加载基因名

geneup <- read.table("easy_input_up.txt")$V1

# 把gene symbol转换为ENTREZ ID

# 此处物种是人，其他物种的ID转换方法，请参考FigureYa52GOplot

geneup.id <- bitr(geneup, fromType = "SYMBOL", toType = "ENTREZID", OrgDb = "org.Hs.eg.db")

# 富集分析

em_ORAup <- setNames(lapply(gmts, enrich_func, geneup.id$ENTREZID, readable = TRUE), names(gmts)) %>%

merge_result2(output = "enrichResult")

# 输出到文件

write.csv(em_ORAup@result, "output_ORA_up.csv", quote = F)

#下调表达的基因也是一样的操作

genedown <- read.table("easy_input_down.txt")$V1

genedown.id <- bitr(genedown, fromType = "SYMBOL", toType = "ENTREZID", OrgDb = "org.Hs.eg.db")

em_ORAdown <- setNames(lapply(gmts, enrich_func, genedown.id$ENTREZID, readable = TRUE), names(gmts)) %>%

merge_result2(output = "enrichResult")

# 上调表达基因的富集结果

pdf(file="ORA_up_DEG.pdf",width = 10,height = 8)

em_plot(em_ORAup, showCategory = 3, fill = "Category", hjust = 10) + scale_fill_brewer(palette="Set1")

dev.off()

# 下调表达基因的富集结果

pdf(file="ORA_down_DEG.pdf",width = 10,height = 8)

em_plot(em_ORAdown, showCategory = 3, fill = "Category", hjust = 5) + scale_fill_brewer(palette="Set1")

dev.off()

# 上调的画在右侧，下调的画在左侧

pdf(file="ORA_ALL_DEG.pdf",width = 10,height = 8)

em_plot(em_ORAup, em_ORAdown, showCategory = 3, fill = "Category", hjust = 12) + scale_fill_brewer(palette="Set1")

dev.off()

# 每种注释筛选2个term

keep1 <- keep_category(em_ORAup, 2)

keep2 <- keep_category(em_ORAdown, 2)

em_ora1 <- em_ORAup[keep1, ]

em_ora2 <- em_ORAdown[keep2, ]

# 上调画右边，下调画左边

em_ora2$Count <- -em_ora2$Count

em_ora <- new("enrichResult",

result = rbind(em_ora1, em_ora2))

# 画barplot

#barplot(em_ora, showCategory = nrow(em_ora)) +

# scale_x_continuous(label = abs) +

# facet_grid(Category ~ ., scales="free",space="free")

# 画dotplot

pdf(file="ORA_ALL_DEG_doplot.pdf",width = 12,height = 8)

dotplot(em_ora, showCategory = nrow(em_ora)) +

scale_x_continuous(label = abs) +

facet_grid(Category ~ ., scales="free",space="free")

dev.off()

#########差异分析

setwd("J:\\免疫相关lncRNA\\脓毒症修回")

rt1=read.table("symbol2.txt",sep="\t",header=T,check.names=F)

rt1 <- as.matrix(rt1)

rownames(rt1)=rt1[,1]

exp=rt1[,2:ncol(rt1)]

dimnames=list(rownames(exp),colnames(exp))

data=matrix(as.numeric(as.matrix(exp)),nrow=nrow(exp),dimnames=dimnames)

data=2^(data)-1

############DEG

data=avereps(data)

data=data[rowMeans(data)>1,]

foldChange=1

padj=0.05

nonmetastasis = 337

metastasis = 63

rt=data #改成自己的文件名

#group=c("normal","tumor","tumor","normal","tumor")

group=c(rep("Non-metastasis",nonmetastasis),rep("Metastasis",metastasis)) #按照癌症和正常样品数目修改

design <- model.matrix(~group)

y <- DGEList(counts=rt,group=group)

y <- calcNormFactors(y)

y <- estimateCommonDisp(y)

y <- estimateTagwiseDisp(y)

et <- exactTest(y,pair = c("Non-metastasis","Metastasis"))

topTags(et)

ordered_tags <- topTags(et, n=100000)

allDiff=ordered_tags$table

allDiff=allDiff[is.na(allDiff$FDR)==FALSE,]

diff=allDiff

newData=y$pseudo.counts

write.table(diff,file="edgerOut.xls",sep="\t",quote=F)

diffSig = diff[(diff$FDR < padj & (diff$logFC>foldChange | diff$logFC<(-foldChange))),]

write.table(diffSig, file="diffSig.xls",sep="\t",quote=F)

diffUp = diff[(diff$FDR < padj & (diff$logFC>foldChange)),]

write.table(diffUp, file="up.xls",sep="\t",quote=F)

diffDown = diff[(diff$FDR < padj & (diff$logFC<(-foldChange))),]

write.table(diffDown, file="down.xls",sep="\t",quote=F)

normalizeExp=rbind(id=colnames(newData),newData)

write.table(normalizeExp,file="normalizeExp.txt",sep="\t",quote=F,col.names=F) #输出所有基因校正后的表达值（normalizeExp.txt）

heatmapData <- newData[rownames(diffSig),]

write.table(heatmapData,file="diffmRNAExp.txt",sep="\t",quote=F)

###################

#colnames(allDiff)[5]= "FDR"

colnames(allDiff)[5]= "FDR"

allDiff$FDR[allDiff$FDR<=0]=2.22329540628561e-322

#volcano

#plot_mode <- "classic" #经典版

plot_mode <- "advanced" #酷炫版

logFCcut <- 0.58 #log2-foldchange

pvalCut <- 0.05 #P.value

adjPcut <- 0.05 #adj.P.value

#for advanced mode

logFCcut2 <- 1

logFCcut3 <- 1.5

pvalCut2 <- 0.00000000000000000001

pvalCut3 <- 0.000000000000000000000000000000000001

#置x，y軸的最大最小位置

xmin <- (range(allDiff$logFC)[1]- (range(allDiff$logFC)[1]+ 20))

xmax <- (range(allDiff$logFC)[1]+ (20-range(allDiff$logFC)[1]))

ymin <- 0

ymax <- 330

# 基因名的颜色，需大于等于pathway的数量，这里自定义了足够多的颜色

mycol <- c("darkgreen","chocolate4","blueviolet","#223D6C","#D20A13","#088247","#58CDD9","#7A142C","#5D90BA","#431A3D","#91612D","#6E568C","#E0367A","#D8D155","#64495D","#7CC767")

###########plot

if (plot_mode == "classic"){

# 簡單的setting for color

allDiff$color_transparent <- ifelse((allDiff$FDR < pvalCut & allDiff$logFC > logFCcut), "red", ifelse((allDiff$FDR < pvalCut & allDiff$logFC < -logFCcut), "blue","grey"))

# 簡單的setting for size

size <- ifelse((allDiff$FDR < pvalCut & abs(allDiff$logFC) > logFCcut), 4, 2)

} else if (plot_mode == "advanced") {

# 複雜的的setting for color

n1 <- length(allDiff[, 1])

cols <- rep("grey", n1)

names(cols)<- rownames(allDiff)

#不同阈值的点的颜色

cols[allDiff$FDR < pvalCut & allDiff$logFC >logFCcut]<- "#FB9A99"

cols[allDiff$FDR < pvalCut2 & allDiff$logFC > logFCcut2]<- "#ED4F4F"

cols[allDiff$FDR < pvalCut & allDiff$logFC < -logFCcut]<- "#B2DF8A"

cols[allDiff$FDR < pvalCut2 & allDiff$logFC < -logFCcut2]<- "#329E3F"

color_transparent <- adjustcolor(cols, alpha.f = 0.5)

allDiff$color_transparent <- color_transparent

# 複雜的的setting for size

n1 <- length(allDiff[, 1])

size <- rep(1, n1)

#不同阈值的点的大小

size[allDiff$FDR < pvalCut & allDiff$logFC > logFCcut]<- 1

size[allDiff$FDR < pvalCut2 & allDiff$logFC > logFCcut2]<- 2

size[allDiff$FDR < pvalCut3 & allDiff$logFC > logFCcut3]<- 3

size[allDiff$FDR < pvalCut & allDiff$logFC < -logFCcut]<- 1

size[allDiff$FDR < pvalCut2 & allDiff$logFC < -logFCcut2]<- 2

size[allDiff$FDR < pvalCut3 & allDiff$logFC < -logFCcut3]<- 3

} else {

stop("Unsupport mode")

}

# Construct the plot object

p1 <- ggplot(data=allDiff, aes(logFC, -log10(FDR))) +

geom_point(alpha = 0.6, size = size, colour = allDiff$color_transparent) +

labs(allDiff=bquote(~Log[2]~"(fold change)"), y=bquote(~-Log[10]~italic("P-value")), title="") +

ylim(c(ymin,ymax)) +

scale_x_continuous(

breaks = c(-10, -5, -logFCcut, 0, logFCcut, 5, 10), #刻度线的位置

labels = c(-10, -5, -logFCcut, 0, logFCcut, 5, 10),

limits = c(-11, 11) #x轴范围，两侧对称才好看

) +

#或用下面这行：

xlim(c(xmin, xmax)) +

#画阈值分界线

geom_vline(xintercept = c(-logFCcut, logFCcut), color="grey40",

linetype="longdash", lwd = 0.5) + #虚线的形状和粗细

geom_hline(yintercept = -log10(pvalCut), color="grey40",

linetype="longdash", lwd = 0.5) +

theme_bw(base_size = 12#, base_family = "Times" #修改字体

) +

theme(panel.grid=element_blank())

if (plot_mode == "advanced") {

p1 <- p1 +

geom_vline(xintercept = c(-logFCcut2, logFCcut2), color="grey40",

linetype="longdash", lwd = 0.5) +

geom_hline(yintercept = -log10(pvalCut2), color="grey40",

linetype="longdash", lwd = 0.5)

}

pdf(file="vol.pdf",5,5)

p1

dev.off()

############

Type=read.table("clinical.txt",sep="\t",header=T,check.names=F,row.names = 1)

Type=as.data.frame(Type)

hmExp=heatmapData

hmExp=log2(hmExp+1)

plotdata = t(hmExp)

# rainbow是R的一个函数，用于产生彩虹色

E = normalizeBetweenArrays(plotdata, method="quantile")

plotdata = E

pdf(file="heatmap_initial.pdf",height=7.5,width=10)

pheatmap(t(plotdata),

annotation=Type,

color = colorRampPalette(c("#3F007D", "white", "#E34A33"))(50),

cluster_cols =F,

cluster_rows = T,

show_colnames = F,

show_rownames = F,

fontsize = 12,

fontsize_row=8,

fontsize_col=10)

dev.off()

#############

library(clusterProfiler)

library(msigdbr)

library(DOSE)

library(enrichplot)

library(ggplot2)

library(plyr)

library(dplyr)

gsym.fc = diff

gsym.fc[1:3,]

#rownames(gsym.fc) = gsym.fc$id

# cutoff

logFCcut <- 1 #log2-foldchange

adjPcut <- 0.05 #adj.P.value

# 筛选差异基因

geneup <- rownames(gsym.fc[gsym.fc$logFC > logFCcut & gsym.fc$FDR < adjPcut, ])

genedown <- rownames(gsym.fc[gsym.fc$logFC < -logFCcut & gsym.fc$FDR < adjPcut, ])

# 数量

length(geneup)

# 保存到文件，便于套用格式

write.table(geneup, "easy_input_up.txt", quote = F, row.names = F, col.names = F)

write.table(genedown, "easy_input_down.txt", quote = F, row.names = F, col.names = F)

#ORA富集分析

# 自定义函数

enrich_func <- function(x, gene, readable = FALSE) {

en_result <- enricher(gene, TERM2GENE = x)

if (readable & nrow(en_result) > 0)

en_result <- setReadable(en_result, 'org.Hs.eg.db', #物种

'ENTREZID')

return(en_result)

}

## 数据处理函数

merge_result2 <- function(enrichResultList, output = "compareClusterResult") {

if ( !is(enrichResultList, "list")) {

stop("input should be a name list...")

}

if ( is.null(names(enrichResultList))) {

stop("input should be a name list...")

}

x <- lapply(enrichResultList, as.data.frame)

names(x) <- names(enrichResultList)

y <- ldply(x, "rbind")

if (output == "compareClusterResult") {

y <- plyr::rename(y, c(.id="Cluster"))

y$Cluster = factor(y$Cluster, levels=names(enrichResultList))

return(new("compareClusterResult",

compareClusterResult = y))

}

y <- plyr::rename(y, c(.id="Category"))

if (output == "enrichResult") {

return(new("enrichResult",

result = y))

}

if (output == "gseaResult") {

return(new("gseaResult",

result = y))

}

}

keep_category <- function(em_ORA, n) {

table_em <- as.numeric(table(em_ORA$Category))

start <- rep(0, length(table_em) - 1)

for(i in seq_len(length(table_em) - 1)) {

start[i] <- sum(table_em[seq_len(i)])

}

showCategorys <- sapply(table_em, function(x) min(n, x))

start <- c(0, start) + 1

end <- start + showCategorys - 1

keep <- NULL

for(i in seq_len(length(start))) {

keep <- c(keep, c(start[i] : end[i]))

}

return(keep)

}

enrich_filter <- function(em_result, showCategory) {

keep <- keep_category(em_result, showCategory)

em_result <- em_result[keep, ]

if ("NES" %in% colnames(em_result))

em_result$Count <- em_result$core_enrichment %>%

strsplit(split = "/") %>%

vapply(length, FUN.VALUE = 1)

return(em_result)

}

## 作图函数

em_plot <- function(em_1 = NULL, em_2 = NULL, showCategory = 2, fill = "p.adjust", hjust = 1) {

fill <- match.arg(fill, c("Category", "p.adjust", "log10_p.adjust"))

result1 <- enrich_filter(em_1, showCategory)

if (is.null(em_2)) {

result <- result1

} else {

result2 <- enrich_filter(em_2, showCategory)

result2$Count <- -result2$Count

result <- rbind(result1, result2)

}

result$Category <- gsub("\n.*", "", result$Category)

result$log10_p.adjust <- log10(result$p.adjust)

data_plot <- result[, c("ID", "Category", "p.adjust", "log10_p.adjust", "Count")]

data_plot2 <- data_plot

data_plot2$ID <- factor(data_plot2$ID, levels = unique(data_plot2$ID))

data_plot2 <- plyr::rename(data_plot2, c("Count" = "gene_number"))

h_just <- ifelse(data_plot2$gene_number < 0, -hjust, hjust)

ggplot(data_plot2, aes_string(x = "gene_number", y = "ID", fill = fill)) +

geom_col() +

geom_text(aes_(x =~ gene_number + h_just, label =~ abs(gene_number)),

color="black") +

scale_x_continuous(label = abs,

expand = expansion(mult = c(.01, .01))) + #两侧留空

theme_classic() +

ylab("") +

theme(axis.title.x = element_text(size = 15)) +

facet_grid(Category ~ ., scales="free", space="free")

}

# 以人为例

#msigdbr_species()

gmt <- msigdbr(species = "Homo sapiens")

gmt2 <- gmt%>%

dplyr::select(gs_name, entrez_gene)

gmts <- split(gmt2, gmt$gs_cat)

# 先以上调表达的基因为例

# 加载基因名

geneup <- read.table("easy_input_up.txt")$V1

# 把gene symbol转换为ENTREZ ID

# 此处物种是人，其他物种的ID转换方法，请参考FigureYa52GOplot

geneup.id <- bitr(geneup, fromType = "SYMBOL", toType = "ENTREZID", OrgDb = "org.Hs.eg.db")

# 富集分析

em_ORAup <- setNames(lapply(gmts, enrich_func, geneup.id$ENTREZID, readable = TRUE), names(gmts)) %>%

merge_result2(output = "enrichResult")

# 输出到文件

write.csv(em_ORAup@result, "output_ORA_up.csv", quote = F)

#下调表达的基因也是一样的操作

genedown <- read.table("easy_input_down.txt")$V1

genedown.id <- bitr(genedown, fromType = "SYMBOL", toType = "ENTREZID", OrgDb = "org.Hs.eg.db")

em_ORAdown <- setNames(lapply(gmts, enrich_func, genedown.id$ENTREZID, readable = TRUE), names(gmts)) %>%

merge_result2(output = "enrichResult")

# 上调表达基因的富集结果

pdf(file="ORA_up_DEG.pdf",width = 10,height = 8)

em_plot(em_ORAup, showCategory = 3, fill = "Category", hjust = 10) + scale_fill_brewer(palette="Set1")

dev.off()

# 下调表达基因的富集结果

pdf(file="ORA_down_DEG.pdf",width = 10,height = 8)

em_plot(em_ORAdown, showCategory = 3, fill = "Category", hjust = 5) + scale_fill_brewer(palette="Set1")

dev.off()

# 上调的画在右侧，下调的画在左侧

pdf(file="ORA_ALL_DEG.pdf",width = 10,height = 8)

em_plot(em_ORAup, em_ORAdown, showCategory = 3, fill = "Category", hjust = 12) + scale_fill_brewer(palette="Set1")

dev.off()

# 每种注释筛选2个term

keep1 <- keep_category(em_ORAup, 2)

keep2 <- keep_category(em_ORAdown, 2)

em_ora1 <- em_ORAup[keep1, ]

em_ora2 <- em_ORAdown[keep2, ]

# 上调画右边，下调画左边

em_ora2$Count <- -em_ora2$Count

em_ora <- new("enrichResult",

result = rbind(em_ora1, em_ora2))

# 画barplot

#barplot(em_ora, showCategory = nrow(em_ora)) +

# scale_x_continuous(label = abs) +

# facet_grid(Category ~ ., scales="free",space="free")

# 画dotplot

pdf(file="ORA_ALL_DEG_doplot.pdf",width = 12,height = 8)

dotplot(em_ora, showCategory = nrow(em_ora)) +

scale_x_continuous(label = abs) +

facet_grid(Category ~ ., scales="free",space="free")

dev.off()

#################

######immune_gene

##########DEG_keygene

fdrFilter=1

logFCfilter=0

#allDiff=topTable(fit2,adjust='fdr',number=200000)

rt=diff

diffExp=read.table("normalizeExp.txt",sep="\t",header=T,check.names=F)

diffExp <- as.matrix(diffExp)

rownames(diffExp)=diffExp[,1]

exp=diffExp[,2:ncol(diffExp)]

dimnames=list(rownames(exp),colnames(exp))

diffExp=matrix(as.numeric(as.matrix(exp)),nrow=nrow(exp),dimnames=dimnames)

gene=read.table("lnc_sig.txt",sep="\t",header=T)

immuneDiffAll=rt[intersect(gene[,1],rownames(rt)),]

immuneDiffGene=intersect(gene[,1],rownames(allDiff))

hmExp=diffExp[which(rownames(diffExp)%in%immuneDiffGene),]

immuneDiffResult=immuneDiffAll[immuneDiffGene,]

immuneDiffResult=rbind(ID=colnames(immuneDiffResult),immuneDiffResult)

write.table(immuneDiffResult,file="Key_Diff.xls",sep="\t",col.names=F,quote=F)

immuneGeneExp=rbind(ID=colnames(hmExp),hmExp)

write.table(immuneGeneExp,file="Key_GeneExp.txt",sep="\t",quote=F,col.names=F)

#volcano

allDiff = immuneDiffAll

colnames(allDiff)[5]= "FDR"

allDiff$FDR[allDiff$FDR<=0]=2.22329540628561e-322

#volcano

#plot_mode <- "classic" #经典版

#plot_mode <- "classic" #经典版

plot_mode <- "advanced" #酷炫版

logFCcut <- 0.58 #log2-foldchange

pvalCut <- 0.05 #P.value

adjPcut <- 0.05 #adj.P.value

#for advanced mode

logFCcut2 <- 1

logFCcut3 <- 1.5

pvalCut2 <- 0.00000000000000000001

pvalCut3 <- 0.000000000000000000000000000000000001

#置x，y軸的最大最小位置

xmin <- (range(allDiff$logFC)[1]- (range(allDiff$logFC)[1]+ 20))

xmax <- (range(allDiff$logFC)[1]+ (20-range(allDiff$logFC)[1]))

ymin <- 0

ymax <- 330

# 基因名的颜色，需大于等于pathway的数量，这里自定义了足够多的颜色

mycol <- c("darkgreen","chocolate4","blueviolet","#223D6C","#D20A13","#088247","#58CDD9","#7A142C","#5D90BA","#431A3D","#91612D","#6E568C","#E0367A","#D8D155","#64495D","#7CC767")

###########plot

if (plot_mode == "classic"){

# 簡單的setting for color

allDiff$color_transparent <- ifelse((allDiff$FDR < pvalCut & allDiff$logFC > logFCcut), "red", ifelse((allDiff$FDR < pvalCut & allDiff$logFC < -logFCcut), "blue","grey"))

# 簡單的setting for size

size <- ifelse((allDiff$FDR < pvalCut & abs(allDiff$logFC) > logFCcut), 4, 2)

} else if (plot_mode == "advanced") {

# 複雜的的setting for color

n1 <- length(allDiff[, 1])

cols <- rep("grey", n1)

names(cols)<- rownames(allDiff)

#不同阈值的点的颜色

cols[allDiff$FDR < pvalCut & allDiff$logFC >logFCcut]<- "#FB9A99"

cols[allDiff$FDR < pvalCut2 & allDiff$logFC > logFCcut2]<- "#ED4F4F"

cols[allDiff$FDR < pvalCut & allDiff$logFC < -logFCcut]<- "#B2DF8A"

cols[allDiff$FDR < pvalCut2 & allDiff$logFC < -logFCcut2]<- "#329E3F"

color_transparent <- adjustcolor(cols, alpha.f = 0.5)

allDiff$color_transparent <- color_transparent

# 複雜的的setting for size

n1 <- length(allDiff[, 1])

size <- rep(1, n1)

#不同阈值的点的大小

size[allDiff$FDR < pvalCut & allDiff$logFC > logFCcut]<- 1

size[allDiff$FDR < pvalCut2 & allDiff$logFC > logFCcut2]<- 2

size[allDiff$FDR < pvalCut3 & allDiff$logFC > logFCcut3]<- 3

size[allDiff$FDR < pvalCut & allDiff$logFC < -logFCcut]<- 1

size[allDiff$FDR < pvalCut2 & allDiff$logFC < -logFCcut2]<- 2

size[allDiff$FDR < pvalCut3 & allDiff$logFC < -logFCcut3]<- 3

} else {

stop("Unsupport mode")

}

# Construct the plot object

p1 <- ggplot(data=allDiff, aes(logFC, -log10(FDR))) +

geom_point(alpha = 0.6, size = size, colour = allDiff$color_transparent) +

labs(allDiff=bquote(~Log[2]~"(fold change)"), y=bquote(~-Log[10]~italic("P-value")), title="") +

ylim(c(ymin,ymax)) +

scale_x_continuous(

breaks = c(-10, -5, -logFCcut, 0, logFCcut, 5, 10), #刻度线的位置

labels = c(-10, -5, -logFCcut, 0, logFCcut, 5, 10),

limits = c(-11, 11) #x轴范围，两侧对称才好看

) +

#或用下面这行：

xlim(c(xmin, xmax)) +

#画阈值分界线

geom_vline(xintercept = c(-logFCcut, logFCcut), color="grey40",

linetype="longdash", lwd = 0.5) + #虚线的形状和粗细

geom_hline(yintercept = -log10(pvalCut), color="grey40",

linetype="longdash", lwd = 0.5) +

theme_bw(base_size = 12#, base_family = "Times" #修改字体

) +

theme(panel.grid=element_blank())

if (plot_mode == "advanced") {

p1 <- p1 +

geom_vline(xintercept = c(-logFCcut2, logFCcut2), color="grey40",

linetype="longdash", lwd = 0.5) +

geom_hline(yintercept = -log10(pvalCut2), color="grey40",

linetype="longdash", lwd = 0.5)

}

pdf(file="vol_keygene.pdf",5,5)

p1

dev.off()

#####heatmap

#Type=c(rep("Primary tumor",nonmetastasis),rep("Metastatic tumor",metastasis))

#Type=c(rep("Primary tumor",nonmetastasis),rep("Bone metastatic tumor",metastasis))

Type=read.table("clinical.txt",sep="\t",header=T,check.names=F,row.names = 1)

hmExp=diffExp[which(rownames(hmExp)%in%immuneDiffGene),]

Type=as.data.frame(Type)

hmExp=log2(hmExp+1)

plotdata = t(hmExp)

# rainbow是R的一个函数，用于产生彩虹色

E = normalizeBetweenArrays(plotdata, method="quantile")

plotdata = E

pdf(file="heatmap_keygene.pdf",height=7.5,width=10)

pheatmap(t(plotdata),

annotation=Type,

color = colorRampPalette(c("#3F007D", "white", "#E34A33"))(50),

cluster_cols =F,

cluster_rows = T,

show_colnames = F,

show_rownames = F,

fontsize = 12,

fontsize_row=8,

fontsize_col=10)

dev.off()

##########DEG_keygene

fdrFilter=0.05

logFCfilter=1

rt=diffSig

diffExp=read.table("normalizeExp.txt",sep="\t",header=T,check.names=F)

diffExp <- as.matrix(diffExp)

rownames(diffExp)=diffExp[,1]

exp=diffExp[,2:ncol(diffExp)]

dimnames=list(rownames(exp),colnames(exp))

diffExp=matrix(as.numeric(as.matrix(exp)),nrow=nrow(exp),dimnames=dimnames)

gene=read.table("TF.txt",sep="\t",header=T)

immuneDiffAll=rt[intersect(gene[,1],rownames(rt)),]

immuneDiffGene=intersect(gene[,1],rownames(diffSig))

hmExp=diffExp[immuneDiffGene,]

immuneDiffResult=immuneDiffAll[immuneDiffGene,]

immuneDiffResult=rbind(ID=colnames(immuneDiffResult),immuneDiffResult)

write.table(immuneDiffResult,file="Key_TF.xls",sep="\t",col.names=F,quote=F)

immuneGeneExp=rbind(ID=colnames(hmExp),hmExp)

write.table(immuneGeneExp,file="Key_TF.txt",sep="\t",quote=F,col.names=F)

#volcano

allDiff = immuneDiffAll

colnames(allDiff)[5]= "FDR"

allDiff$FDR[allDiff$FDR<=0]=2.22329540628561e-322

#volcano

#plot_mode <- "classic" #经典版

#plot_mode <- "classic" #经典版

plot_mode <- "advanced" #酷炫版

logFCcut <- 0.58 #log2-foldchange

pvalCut <- 0.05 #P.value

adjPcut <- 0.05 #adj.P.value

#for advanced mode

logFCcut2 <- 1

logFCcut3 <- 1.5

pvalCut2 <- 0.00000000000000000001

pvalCut3 <- 0.000000000000000000000000000000000001

#置x，y軸的最大最小位置

xmin <- (range(allDiff$logFC)[1]- (range(allDiff$logFC)[1]+ 20))

xmax <- (range(allDiff$logFC)[1]+ (20-range(allDiff$logFC)[1]))

ymin <- 0

ymax <- 330

# 基因名的颜色，需大于等于pathway的数量，这里自定义了足够多的颜色

mycol <- c("darkgreen","chocolate4","blueviolet","#223D6C","#D20A13","#088247","#58CDD9","#7A142C","#5D90BA","#431A3D","#91612D","#6E568C","#E0367A","#D8D155","#64495D","#7CC767")

###########plot

if (plot_mode == "classic"){

# 簡單的setting for color

allDiff$color_transparent <- ifelse((allDiff$FDR < pvalCut & allDiff$logFC > logFCcut), "red", ifelse((allDiff$FDR < pvalCut & allDiff$logFC < -logFCcut), "blue","grey"))

# 簡單的setting for size

size <- ifelse((allDiff$FDR < pvalCut & abs(allDiff$logFC) > logFCcut), 4, 2)

} else if (plot_mode == "advanced") {

# 複雜的的setting for color

n1 <- length(allDiff[, 1])

cols <- rep("grey", n1)

names(cols)<- rownames(allDiff)

#不同阈值的点的颜色

cols[allDiff$FDR < pvalCut & allDiff$logFC >logFCcut]<- "#FB9A99"

cols[allDiff$FDR < pvalCut2 & allDiff$logFC > logFCcut2]<- "#ED4F4F"

cols[allDiff$FDR < pvalCut & allDiff$logFC < -logFCcut]<- "#B2DF8A"

cols[allDiff$FDR < pvalCut2 & allDiff$logFC < -logFCcut2]<- "#329E3F"

color_transparent <- adjustcolor(cols, alpha.f = 0.5)

allDiff$color_transparent <- color_transparent

# 複雜的的setting for size

n1 <- length(allDiff[, 1])

size <- rep(1, n1)

#不同阈值的点的大小

size[allDiff$FDR < pvalCut & allDiff$logFC > logFCcut]<- 1

size[allDiff$FDR < pvalCut2 & allDiff$logFC > logFCcut2]<- 2

size[allDiff$FDR < pvalCut3 & allDiff$logFC > logFCcut3]<- 3

size[allDiff$FDR < pvalCut & allDiff$logFC < -logFCcut]<- 1

size[allDiff$FDR < pvalCut2 & allDiff$logFC < -logFCcut2]<- 2

size[allDiff$FDR < pvalCut3 & allDiff$logFC < -logFCcut3]<- 3

} else {

stop("Unsupport mode")

}

# Construct the plot object

p1 <- ggplot(data=allDiff, aes(logFC, -log10(FDR))) +

geom_point(alpha = 0.6, size = size, colour = allDiff$color_transparent) +

labs(allDiff=bquote(~Log[2]~"(fold change)"), y=bquote(~-Log[10]~italic("P-value")), title="") +

ylim(c(ymin,ymax)) +

scale_x_continuous(

breaks = c(-10, -5, -logFCcut, 0, logFCcut, 5, 10), #刻度线的位置

labels = c(-10, -5, -logFCcut, 0, logFCcut, 5, 10),

limits = c(-11, 11) #x轴范围，两侧对称才好看

) +

#或用下面这行：

xlim(c(xmin, xmax)) +

#画阈值分界线

geom_vline(xintercept = c(-logFCcut, logFCcut), color="grey40",

linetype="longdash", lwd = 0.5) + #虚线的形状和粗细

geom_hline(yintercept = -log10(pvalCut), color="grey40",

linetype="longdash", lwd = 0.5) +

theme_bw(base_size = 12#, base_family = "Times" #修改字体

) +

theme(panel.grid=element_blank())

if (plot_mode == "advanced") {

p1 <- p1 +

geom_vline(xintercept = c(-logFCcut2, logFCcut2), color="grey40",

linetype="longdash", lwd = 0.5) +

geom_hline(yintercept = -log10(pvalCut2), color="grey40",

linetype="longdash", lwd = 0.5)

}

pdf(file="vol_TF.pdf",5,5)

p1

dev.off()

#####heatmap

#Type=c(rep("Primary tumor",nonmetastasis),rep("Metastatic tumor",metastasis))

#Type=c(rep("Primary tumor",nonmetastasis),rep("Bone metastatic tumor",metastasis))

Type=read.table("clinical.txt",sep="\t",header=T,check.names=F,row.names = 1)

hmExp=diffExp[which(rownames(hmExp)%in%immuneDiffGene),]

Type=as.data.frame(Type)

hmExp=log2(hmExp+1)

plotdata = t(hmExp)

# rainbow是R的一个函数，用于产生彩虹色

E = normalizeBetweenArrays(plotdata, method="quantile")

plotdata = E

pdf(file="heatmap_TF.pdf",height=7.5,width=10)

pheatmap(t(plotdata),

annotation=Type,

color = colorRampPalette(c("#3F007D", "white", "#E34A33"))(50),

cluster_cols =F,

cluster_rows = T,

show_colnames = F,

show_rownames = F,

fontsize = 12,

fontsize_row=8,

fontsize_col=10)

dev.off()

####################logistic

Type=read.table("clinical.txt",sep="\t",header=T,check.names=F,row.names = 1)######调整样本分组

mRNA=read.table("Key_GeneExp.txt",sep="\t",header=T,check.names=F,row.names = 1)

#??Ʒȡ????

sameSample=intersect(row.names(Type),colnames(mRNA))

Type=Type[sameSample,]

mRNA=mRNA[,sameSample]

mRNA = t(mRNA)

Type=ifelse(Type$Group == c("Normal PBMC") ,0,1)

#?ϲ?????

data=cbind(Type,mRNA)

data=rbind(ID=colnames(data),data)

write.table(data,file="logistic.txt",sep="\t",col.names=F,quote=F)

################

library(limma)

library(pheatmap)

library(caret)

library(pROC)

library(glmnet)

fdrFilter=1 #????????ʱfdr?ٽ?ֵ

foldChange=1 #????????ʱfold change????????

logFCfilter=log2(foldChange) #logFC?ٽ?ֵ

for(i in 1:1000){

####################?????ݽ??з???####################

data=read.table("logistic.txt",header=T,sep="\t",check.names=F,row.names=1) #??ȡ?????ļ?

cli=read.table("clinical.txt",sep="\t",check.names=F,header=T,row.names=1) #??ȡ?ٴ??ļ?

sameSample=intersect(row.names(cli),row.names(data))

data=data[sameSample,]

cli=cli[sameSample,]

#???ݷ???

inTrain=createDataPartition(y=data[,1],p=0.7,list=F) #Train??60%

train=data[inTrain,]

test=data[-inTrain,]

#???????????ݽ???

trainOut=cbind(id=row.names(train),train)

testOut=cbind(id=row.names(test),test)

write.table(trainOut,file="train.txt",sep="\t",quote=F,row.names=F)

write.table(testOut,file="test.txt",sep="\t",quote=F,row.names=F)

#?????????ٴ?????

trainCli=cli[row.names(train),]

testCli=cli[row.names(test),]

trainFlag=cbind(trainCli,flag="Train")

testFlag=cbind(testCli,flag="Test")

newTable=rbind(trainFlag,testFlag)

cliStatOut=data.frame()

for(i in 1:(ncol(newTable)-1)){

nameStat=colnames(newTable)[i]

tableStat=table(newTable[,c(nameStat,"flag")])

tableStatSum=cbind(Total=rowSums(tableStat),tableStat)

tableStatRatio=prop.table(tableStatSum,2)

tableStatRatio=round(tableStatRatio*100,2)

tableStatPaste=paste(tableStatSum,"(",tableStatRatio,"%)",sep="")

tableStatOut=matrix(tableStatPaste,ncol=3,dimnames=dimnames(tableStatSum))

pStat=chisq.test(tableStat[row.names(tableStat)!="unknow",])

pValueStat=round(pStat$p.value,4)

pValueCol=c(pValueStat,rep(" ",(nrow(tableStatOut)-1)) )

tableStatOut=cbind(Covariates=nameStat,Type=row.names(tableStatOut),tableStatOut,Pvalue=pValueCol)

cliStatOut=rbind(cliStatOut,tableStatOut)

}

minPval=min(as.numeric(as.vector(cliStatOut[cliStatOut[,"Pvalue"]!=" ","Pvalue"])))

write.table(cliStatOut,file="cliStat.xls",sep="\t",quote=F,row.names=F)

#??ȡ?????ļ????????????ļ?????

rt=read.table("train.txt",sep="\t",header=T,check.names=F,row.names=1)

rtLow=rt[rt$Type==0,]

rtHigh=rt[rt$Type==1,]

conNum=nrow(rtLow)

treatNum=nrow(rtHigh)

data=rbind(rtLow,rtHigh)

data=t(data[,(2:ncol(data))])

#????????

outTab=data.frame()

grade=c(rep(1,conNum),rep(2,treatNum))

samplePercent=ncol(data)*0.1

for(i in row.names(data)){

#ɾ??0̫????miRNA

if(length(data[i,(data[i,]==0)])>samplePercent){

next

}

geneName=unlist(strsplit(i,"\\|",))[1]

geneName=gsub("\\/", "_", geneName)

#????????

rt=rbind(expression=data[i,],grade=grade)

rt=as.matrix(t(rt))

wilcoxTest<-wilcox.test(expression ~ grade, data=rt)

conGeneMeans=mean(data[i,1:conNum])

treatGeneMeans=mean(data[i,(conNum+1):ncol(data)])

logFC=log2(treatGeneMeans)-log2(conGeneMeans)

pvalue=wilcoxTest$p.value

conMed=median(data[i,1:conNum])

treatMed=median(data[i,(conNum+1):ncol(data)])

diffMed=treatMed-conMed

if( ((logFC>0) & (diffMed>0)) | ((logFC<0) & (diffMed<0)) ){

outTab=rbind(outTab,cbind(gene=i,conMean=conGeneMeans,treatMean=treatGeneMeans,logFC=logFC,pValue=pvalue))

}

}

pValue=outTab[,"pValue"]

fdr=p.adjust(as.numeric(as.vector(pValue)),method="fdr")

outTab=cbind(outTab,fdr=fdr)

#???????л????Ĳ???????

write.table(outTab,file="all.xls",sep="\t",row.names=F,quote=F)

#????????????

outDiff=outTab[( abs(as.numeric(as.vector(outTab$logFC)))>logFCfilter & as.numeric(as.vector(outTab$fdr))<fdrFilter),]

write.table(outDiff,file="diff.xls",sep="\t",row.names=F,quote=F)

#?????????ı????ļ?

heatmap=data[as.vector(outDiff[,1]),]

heatmap=t(heatmap)

heatmap=log2(heatmap+1)

Type=c(rep(0,conNum),rep(1,treatNum))

heatmap=cbind(Type,heatmap)

heatmap=rbind(ID=colnames(heatmap),heatmap)

write.table(heatmap,file="diffMrnaExp.txt",sep="\t",col.names=F,quote=F)

#???Ʋ?????????ͼ

hmExp=data[as.vector(outDiff[,1]),]

hmExp=log2(hmExp+1)

Type=c(rep("untreated",conNum),rep("treated",treatNum))

names(Type)=colnames(data)

Type=as.data.frame(Type)

pdf(file="heatmap_train_deg.pdf",height=6,width=10)

pheatmap(hmExp,

annotation=Type,

color = colorRampPalette(c("#377EB8", "white", "#E41A1C"))(50),

cluster_cols =F,

scale="row",

show_colnames = F,

show_rownames = T,

fontsize = 6,

fontsize_row=6,

fontsize_col=6)

dev.off()

####################ģ?͹???####################

train=read.table("diffMrnaExp.txt",header=T,sep="\t",check.names=F,row.names=1) #??ȡtrain?????ļ?

test=read.table("test.txt",header=T,sep="\t",check.names=F,row.names=1) #??ȡtest?????ļ?

test[,c(2:ncol(test))]=log2(test[,c(2:ncol(test))]+1)

test=test[,colnames(train)]

rt=rbind(train,test)

#????ģ??

x=as.matrix(train[,c(2:ncol(train))])

y=train[,1]

fit=glmnet(x, y, family = "binomial", alpha=1)

cvfit=cv.glmnet(x, y, family="binomial", alpha=1,type.measure='auc',nfolds = 10)

pdf(file="cvfit.pdf",width=6,height=5.5)

plot(cvfit)

dev.off()

#???????ػ???ϵ??

coef=coef(fit, s = cvfit$lambda.min)

index=which(coef != 0)

actCoef=coef[index]

lassoGene=row.names(coef)[index]

coef=cbind(Gene=lassoGene,Coef=actCoef)

write.table(coef,file="coef.txt",sep="\t",quote=F,row.names=F)

write.table(lassoGene[-1],file="mRNAlist.txt",sep="\t",quote=F,row.names=F,col.names=F)

#???庯??

threshold=0.5

bioStat=function(x=null, y=null, dataType=null){

pred=predict(cvfit,newx=x,s=cvfit$lambda.min,type = 'response')

outTab=cbind(id=row.names(pred),Type=as.character(y),pred)

colnames(outTab)=c("id","Type","value")

sameGene=intersect(colnames(x),lassoGene)

outTab=cbind(outTab,x[,sameGene])

write.table(outTab,file=paste0("value.",dataType,".txt"),sep="\t",quote=F,row.names=F)

#ͳ??ֵ

pred_new=as.integer(pred>threshold)

tab=table(pred_new,y)

if((nrow(tab)==2) & (ncol(tab)==2)){

TP=tab[2,2];TN=tab[1,1];FP=tab[2,1];FN=tab[1,2]

Accuracy=round((TP+TN)/(TP+FN+FP+TN),4)

SE=round(TP/(TP+FN),4)

SP=round(TN/(TN+FP),4)

PPV=round(TP/(TP+FP),4)

NPV=round(TN/(TN+FN),4)

#????AUCֵ

rocobj1=roc(y, as.vector(pred))

AUC=auc(rocobj1)

AUC=round(AUC,4)

return(c(SE,SP,PPV,NPV,Accuracy,AUC))

}

}

#train??????

trainValue=bioStat(x=x,y=y,dataType="train")

#test??????

testX=as.matrix(test[,c(2:ncol(test))])

testY=test[,1]

testValue=bioStat(x=testX,y=testY,dataType="test")

#??????Ʒ?Ľ???

totalX=as.matrix(rt[,c(2:ncol(rt))])

totalY=rt[,1]

totalValue=bioStat(x=totalX,y=totalY,dataType="all")

#????????????

statTab=rbind(Train=trainValue,Test=testValue,Total=totalValue)

colnames(statTab)=c("SE","SP","PPV","NPV","Accuracy","AUC")

statTab=rbind(ID=colnames(statTab),statTab)

write.table(statTab,file="accuracyStat.xls",sep="\t",col.names=F,quote=F)

if((length(statTab[statTab<0.70])<=1)&(minPval>0.05)){

break

}else{

file.remove("all.xls")

file.remove("coef.txt")

file.remove("cvfit.pdf")

file.remove("diff.xls")

file.remove("diffMrnaExp.txt")

file.remove("heatmap_logistic.pdf")

file.remove("accuracyStat.xls")

file.remove("test.txt")

file.remove("train.txt")

file.remove("value.all.txt")

file.remove("value.test.txt")

file.remove("value.train.txt")

file.remove("cliStat.xls")

}

}

#######ROC

library(pROC) #???ð?

#????ROC???ߺ???

rocPlot=function(inputFile=null,outPdf=null,mainName=null){

rt=read.table(inputFile,header=T,sep="\t",check.names=F,row.names=1)

rocobj1=roc(rt$Type, as.vector(rt$value))

pdf(file=outPdf,width=4,height=4)

plot(rocobj1, print.auc=TRUE, col="red",main=mainName)

dev.off()

}

#????train??ROC????

rocPlot(inputFile="value.train.txt",outPdf="ROC.train.pdf",mainName="Train set")

#????test??ROC????

rocPlot(inputFile="value.test.txt",outPdf="ROC.test.pdf",mainName="Test set")

#??????????ƷROC????

rocPlot(inputFile="value.all.txt",outPdf="ROC.all.pdf",mainName="Total set")

#train??test??ROC????

train=read.table("value.train.txt",header=T,sep="\t",check.names=F,row.names=1)

trainRoc=roc(train$Type, as.vector(train$value))

test=read.table("value.test.txt",header=T,sep="\t",check.names=F,row.names=1)

testRoc=roc(test$Type, as.vector(test$value))

pdf(file="train_test.pdf",width=4,height=4)

plot(trainRoc, col="#ED0000E5")

lines(testRoc, col="#00468BE5")

aucText=c(paste0("Train set, AUC=",sprintf("%0.3f",auc(trainRoc))),paste0("Test set, AUC=",sprintf("%0.3f",auc(testRoc))))

legend("bottomright", aucText,lwd=2,bty="n",col=c("#ED0000E5","#00468BE5"))

dev.off()

#########PCA

library(ggplot2) #???ð?

#???Ʋ???miRNA??PCAͼ

rt=read.table("diffMrnaExp.txt",sep="\t",header=T,check.names=F,row.names=1) #??ȡ?????ļ?

data=rt[,2:ncol(rt)]

Type=ifelse(rt[,1]==0,"Normal","RA")

#PCA????

data.pca=prcomp(data, scale. = TRUE)

pcaPredict=predict(data.pca)

PCA = data.frame(PC1 = pcaPredict[,1], PC2 = pcaPredict[,2],group=Type)

#???ӻ?

pdf(file="PCA.diff.pdf",height=3,width=4) #???????????ļ?

ggplot(data = PCA, aes(PC1, PC2)) + geom_point(aes(color = Type)) +

theme_bw()+

theme(plot.margin=unit(c(2,1,1,1),'lines'))+

theme(panel.grid.major = element_blank(), panel.grid.minor = element_blank())

dev.off()

#????ģ??miRNA??PCAͼ

rt=read.table("value.train.txt",sep="\t",header=T,check.names=F,row.names=1) #??ȡ?????ļ?

data=rt[,3:ncol(rt)]

Type=ifelse(rt[,1]==0,"Normal","RA")

#PCA????

data.pca=prcomp(data, scale. = TRUE)

pcaPredict=predict(data.pca)

PCA = data.frame(PC1 = pcaPredict[,1], PC2 = pcaPredict[,2],group=Type)

#???ӻ?

pdf(file="PCA.model.pdf",height=3,width=4) #???????????ļ?

ggplot(data = PCA, aes(PC1, PC2)) + geom_point(aes(color = Type)) +

theme_bw()+

theme(plot.margin=unit(c(2,1,1,1),'lines'))+

theme(panel.grid.major = element_blank(), panel.grid.minor = element_blank())

dev.off()

#########ssGSEA

#############imm_ssGSEA

library(reshape2)

library(ggpubr)

library(limma)

library(GSEABase)

library(GSVA)

expFile="Group_symbol_FPKM.txt" #?????????ļ?

gmtFile="J:\\metastasis_scRNA_seq\\RNA-seq\\logistics\\immune.gmt" #???????ݼ??ļ?

#??ȡ?????????ļ?,?????????ļ?????

rt=read.table(expFile, header=T, sep="\t", check.names=F)

rt=as.matrix(rt)

rownames(rt)=rt[,1]

exp=rt[,2:ncol(rt)]

dimnames=list(rownames(exp),colnames(exp))

data=matrix(as.numeric(as.matrix(exp)),nrow=nrow(exp),dimnames=dimnames)

data=avereps(data)

data[1:5,1:5]

#??ȡ???????ļ?

geneSets=getGmt(gmtFile, geneIdType=SymbolIdentifier())

#ssGSEA????

ssgseaScore=gsva(data, geneSets, method='ssgsea', kcdf='Gaussian', abs.ranking=TRUE)

#??ssGSEA???ֽ??н???

normalize=function(x){

return((x-min(x))/(max(x)-min(x)))}

ssgseaScore=normalize(ssgseaScore)

#????ssGSEA???ֽ???

ssgseaOut=rbind(id=colnames(ssgseaScore), ssgseaScore)

write.table(ssgseaOut,file="ssgseaOut.txt",sep="\t",quote=F,col.names=F)

rt=read.table("ssgseaOut.txt",sep="\t",header=T,check.names=F,row.names = 1)

library(pheatmap)

Type=read.table("clinical.txt",sep="\t",header=T,check.names=F,row.names = 1)

#Type=c(rep("normal whole blood",nonmetastasis),rep("Septic shock blood",metastasis))

#Type=c(rep("non-metastasis",nonmetastasis),rep("metastasis",metastasis))

#Type=c(rep("primarytumor",nonmetastasis),rep("new_tumor_event",metastasis))

Type=as.data.frame(Type)

plotdata = t(rt)

# rainbow是R的一个函数，用于产生彩虹色

E = normalizeBetweenArrays(plotdata, method="quantile")

plotdata = E

pdf(file="heatmap_ssgsea.pdf", width = 6,height =8)

pheatmap(t(plotdata),

annotation=Type,

color = colorRampPalette(c("#377EB8", "white", "#E41A1C"))(50),

cluster_cols =F,

fontsize = 6,

show_colnames = F,

fontsize_row=8,

fontsize_col=4)

dev.off()

#######################

##########GSVA

load("J:\\新课程\\小丫画图\\GSVA\\hallmark.gs.RData")

library(GSVA)

library(limma)

library(GSEABase)

rt=read.table("Group_symbol_FPKM.txt",sep="\t",header=T,check.names=F)

rt <- as.matrix(rt)

rownames(rt)=rt[,1]

exp=rt[,2:ncol(rt)]

dimnames=list(rownames(exp),colnames(exp))

gsym.expr=matrix(as.numeric(as.matrix(exp)),nrow=nrow(exp),dimnames=dimnames)

head(gsym.expr)

gsva_es <- gsva(as.matrix(gsym.expr), gs)

write.csv(gsva_es, "gsva_out.csv", quote = F)

# differential analysis

logFCcutoff=0.0001

adjPvalueCutoff=0.05

group_list <- data.frame(sample = colnames(gsva_es), group = c(rep("Nonmetastasis", nonmetastasis), rep("Metastasis", metastasis)))

head(group_list)

# 设置对比

design <- model.matrix(~ 0 + factor(group_list$group))

colnames(design) <- levels(factor(group_list$group))

rownames(design) <- colnames(gsva_es)

# 构建差异比较矩阵

contrast.matrix <- makeContrasts(Nonmetastasis, levels = design)

# 差异分析，b vs. a

fit <- lmFit(gsva_es, design)

fit2 <- contrasts.fit(fit, contrast.matrix)

fit2 <- eBayes(fit2)

x <- topTable(fit2, coef = 1, n = Inf, adjust.method = "BH", sort.by = "P")

head(x)

#把通路的limma分析结果保存到文件

write.csv(x, "gsva_limma.csv", quote = F)

#输出t值，用做FigureYa39bar的输入数据

pathway <- str_replace(row.names(x), "HALLMARK_", "")

df <- data.frame(ID = pathway, score = -x$t)

write.csv(df, "easy_input2_for39bar.csv", quote = F, row.names = F)

df <- read.csv("easy_input2_for39bar.csv")

head(df)

#按照score的值分组

cutoff <- 1

df$group <- cut(df$score, breaks = c(-Inf, -cutoff, cutoff, Inf),labels = c(1,2,3))

#按照score排序

sortdf <- df[order(df$score),]

sortdf$ID <- factor(sortdf$ID, levels = sortdf$ID)

head(sortdf)

pdf("gsva.pdf", width = 9, height = 12)

ggplot(sortdf, aes(ID, score, fill = group)) + geom_bar(stat = 'identity') +

coord_flip() +

scale_fill_manual(values = c("#00AFBB", 'snow3', "#E7B800"), guide = FALSE) +

#画2条虚线

geom_hline(yintercept = c(-cutoff,cutoff),

color="white",

linetype = 2, #画虚线

size = 0.3) + #线的粗细

#写label

geom_text(data = subset(df, score > 0),

aes(x=ID, y= -0.1, label= paste0(" ", ID), color = group),#bar跟坐标轴间留出间隙

size = 3, #字的大小

hjust = "outward" ) + #字的对齐方式

geom_text(data = subset(df, score < 0),

aes(x=ID, y= 0.1, label=ID, color = group),

size = 3, hjust = "inward") +

scale_colour_manual(values = c("black","snow3","black"), guide = FALSE) +

xlab("") +ylab("t value of GSVA score")+

theme_bw() + #去除背景色

theme(panel.grid =element_blank()) + #去除网格线

theme(panel.border = element_rect(size = 0.6)) + #边框粗细

theme(axis.line.y = element_blank(), axis.ticks.y = element_blank(), axis.text.y = element_blank()) #去除y轴

dev.off()

######vol

#输出t值，用做FigureYa39bar的输入数据

pathway <- str_replace(row.names(x), "HALLMARK_", "")

rownames(x) <- pathway

allDiff=x

allDiff$logFC=-allDiff$logFC

plot_mode <- "advanced" #酷炫版

logFCcut <- 0.01 #log2-foldchange

pvalCut <- 0.05 #P.value

adjPcut <- 0.05 #adj.P.value

#for advanced mode

logFCcut2 <- 0.1

logFCcut3 <- 0.2

pvalCut2 <- 0.01

pvalCut3 <- 0.001

#置x，y軸的最大最小位置

xmin <- (range(allDiff$logFC)[1]- (range(allDiff$logFC)[1]+ 0.4))

xmax <- (range(allDiff$logFC)[1]+ (0.4-range(allDiff$logFC)[1]))

ymin <- 0

ymax <- -log10(allDiff$P.Value)[1] * 1.2

# 基因名的颜色，需大于等于pathway的数量，这里自定义了足够多的颜色

mycol <- c("darkgreen","chocolate4","blueviolet","#223D6C","#D20A13","#088247","#58CDD9","#7A142C","#5D90BA","#431A3D","#91612D","#6E568C","#E0367A","#D8D155","#64495D","#7CC767")

###########plot

if (plot_mode == "classic"){

# 簡單的setting for color

allDiff$color_transparent <- ifelse((allDiff$P.Value < pvalCut & allDiff$logFC > logFCcut), "red", ifelse((allDiff$P.Value < pvalCut & allDiff$logFC < -logFCcut), "blue","grey"))

# 簡單的setting for size

size <- ifelse((allDiff$P.Value < pvalCut & abs(allDiff$logFC) > logFCcut), 4, 2)

} else if (plot_mode == "advanced") {

# 複雜的的setting for color

n1 <- length(allDiff[, 1])

cols <- rep("grey", n1)

names(cols)<- rownames(allDiff)

#不同阈值的点的颜色

cols[allDiff$P.Value < pvalCut & allDiff$logFC >logFCcut]<- "#FB9A99"

cols[allDiff$P.Value < pvalCut2 & allDiff$logFC > logFCcut2]<- "#ED4F4F"

cols[allDiff$P.Value < pvalCut & allDiff$logFC < -logFCcut]<- "#B2DF8A"

cols[allDiff$P.Value < pvalCut2 & allDiff$logFC < -logFCcut2]<- "#329E3F"

color_transparent <- adjustcolor(cols, alpha.f = 0.5)

allDiff$color_transparent <- color_transparent

# 複雜的的setting for size

n1 <- length(allDiff[, 1])

size <- rep(1, n1)

#不同阈值的点的大小

size[allDiff$P.Value < pvalCut & allDiff$logFC > logFCcut]<- 1.5

size[allDiff$P.Value < pvalCut2 & allDiff$logFC > logFCcut2]<- 3

size[allDiff$P.Value < pvalCut3 & allDiff$logFC > logFCcut3]<- 4.5

size[allDiff$P.Value < pvalCut & allDiff$logFC < -logFCcut]<- 1.5

size[allDiff$P.Value < pvalCut2 & allDiff$logFC < -logFCcut2]<- 3

size[allDiff$P.Value < pvalCut3 & allDiff$logFC < -logFCcut3]<- 4.5

} else {

stop("Unsupport mode")

}

# Construct the plot object

p1 <- ggplot(data=allDiff, aes(logFC, -log10(P.Value))) +

geom_point(alpha = 0.6, size = size, colour = allDiff$color_transparent) +

labs(allDiff=bquote(~Log[2]~"(fold change)"), y=bquote(~-Log[10]~italic("P-value")), title="") +

ylim(c(ymin,ymax)) +

#scale_x_continuous(

# breaks = c(-10, -5, -logFCcut, 0, logFCcut, 5, 10), #刻度线的位置

#labels = c(-10, -5, -logFCcut, 0, logFCcut, 5, 10),

#limits = c(-11, 11) #x轴范围，两侧对称才好看

#) +

#或用下面这行：

xlim(c(xmin, xmax)) +

#画阈值分界线

geom_vline(xintercept = c(-logFCcut, logFCcut), color="grey40",

linetype="longdash", lwd = 0.5) + #虚线的形状和粗细

geom_hline(yintercept = -log10(pvalCut), color="grey40",

linetype="longdash", lwd = 0.5) +

theme_bw(base_size = 12#, base_family = "Times" #修改字体

) +

theme(panel.grid=element_blank())

if (plot_mode == "advanced") {

p1 <- p1 +

geom_vline(xintercept = c(-logFCcut2, logFCcut2), color="grey40",

linetype="longdash", lwd = 0.5) +

geom_hline(yintercept = -log10(pvalCut2), color="grey40",

linetype="longdash", lwd = 0.5)

}

pdf(file="vol_HM.pdf", width = 4,height =4)

p1

dev.off()

##########heatmap

diff <- topTable(fit2, coef = 1, n = Inf, adjust.method = "BH", sort.by = "P", p.value=adjPvalueCutoff)

diffName=row.names(diff)

write.table(diff,file="diff_HM.xls",sep="\t",quote=F,col.names=F)

hmExp=gsva_es[diffName,]

hmExp=rbind(id=colnames(hmExp),hmExp)

write.table(hmExp,file="heatmap_HM.txt",sep="\t",quote=F,col.names=F)

rt=gsva_es

library(pheatmap)

Type=read.table("clinical.txt",sep="\t",header=T,check.names=F,row.names = 1)

Type=as.data.frame(Type)

plotdata = t(rt)

# rainbow是R的一个函数，用于产生彩虹色

E = normalizeBetweenArrays(plotdata, method="quantile")

plotdata = E

pdf(file="heatmap_HM.pdf", width = 10,height =8)

pheatmap(t(plotdata),

annotation=Type,

color = colorRampPalette(c("#3F007D", "white", "#E34A33"))(50),

cluster_cols =F,

fontsize = 6,

show_colnames = F,

fontsize_row=8,

fontsize_col=4)

dev.off()

####################

#########cor GSVA

corFilter=0.40

pvalueFilter=0.05

TF = read.table("Key_GeneExp.txt", row.names=1 ,header=T,sep="\t",check.names=F)

uniSigExp = read.table("mRNAlist.txt", header=F,sep="\t",check.names=F)

gene1 = as.vector(uniSigExp[,1])

TF= TF[gene1,]

immuneGene = gsva_es

sameSample=intersect(colnames(TF),colnames(immuneGene))

TF1=TF[,sameSample]

immuneGene1=immuneGene[,sameSample]

outTab=data.frame()

for(i in row.names(TF1)){

if(sd(TF1[i,])>0){

for(j in row.names(immuneGene1)){

x=as.numeric(TF1[i,])

y=as.numeric(immuneGene1[j,])

corT=cor.test(x,y)

cor=corT$estimate

pvalue=corT$p.value

if((cor>corFilter) & (pvalue<pvalueFilter)){

outTab=rbind(outTab,cbind(TF=i,immuneGene=j,cor,pvalue,Regulation="postive"))

}

if((cor< -corFilter) & (pvalue<pvalueFilter)){

outTab=rbind(outTab,cbind(TF=i,immuneGene=j,cor,pvalue,Regulation="negative"))

}

}

}

}

write.table(file="corResult_GSVA.txt",outTab,sep="\t",quote=F,row.names=F)

dim(outTab)

#########cor TF

corFilter=0.60

pvalueFilter=0.05

TF = read.table("Key_GeneExp.txt", row.names=1 ,header=T,sep="\t",check.names=F)

uniSigExp = read.table("mRNAlist.txt", header=F,sep="\t",check.names=F)

gene1 = as.vector(uniSigExp[,1])

TF= TF[gene1,]

immuneGene =read.table("Key_TF.txt", row.names=1 ,header=T,sep="\t",check.names=F)

sameSample=intersect(colnames(TF),colnames(immuneGene))

TF1=TF[,sameSample]

immuneGene1=immuneGene[,sameSample]

outTab=data.frame()

for(i in row.names(TF1)){

if(sd(TF1[i,])>0){

for(j in row.names(immuneGene1)){

x=as.numeric(TF1[i,])

y=as.numeric(immuneGene1[j,])

corT=cor.test(x,y)

cor=corT$estimate

pvalue=corT$p.value

if((cor>corFilter) & (pvalue<pvalueFilter)){

outTab=rbind(outTab,cbind(TF=i,immuneGene=j,cor,pvalue,Regulation="postive"))

}

if((cor< -corFilter) & (pvalue<pvalueFilter)){

outTab=rbind(outTab,cbind(TF=i,immuneGene=j,cor,pvalue,Regulation="negative"))

}

}

}

}

write.table(file="corResult_TF.txt",outTab,sep="\t",quote=F,row.names=F)

dim(outTab)

########ssgasa

corFilter=0.40

pvalueFilter=0.05

TIMER = read.table("ssgseaOut.txt", row.names=1 ,header=T,sep="\t",check.names=F)

immuneGene = read.table("Key_GeneExp.txt", row.names=1 ,header=T,sep="\t",check.names=F)

uniSigExp = read.table("mRNAlist.txt", header=F,sep="\t",check.names=F)

gene1 = as.vector(uniSigExp[,1])

immuneGene= immuneGene[gene1,]

sameSample=intersect(colnames(TIMER),colnames(immuneGene))

TIMER=TIMER[,sameSample]

immuneGene1=immuneGene[,sameSample]

outTab=data.frame()

for(i in row.names(TIMER)){

if(sd(TIMER[i,])>0){

for(j in row.names(immuneGene1)){

x=as.numeric(TIMER[i,])

y=as.numeric(immuneGene1[j,])

corT=cor.test(x,y)

cor=corT$estimate

pvalue=corT$p.value

if((cor>corFilter) & (pvalue<pvalueFilter)){

outTab=rbind(outTab,cbind(TF=i,immuneGene=j,cor,pvalue,Regulation="postive"))

}

if((cor< -corFilter) & (pvalue<pvalueFilter)){

outTab=rbind(outTab,cbind(TF=i,immuneGene=j,cor,pvalue,Regulation="negative"))

}

}

}

}

write.table(file="corResult_ssGSEA.txt",outTab,sep="\t",quote=F,row.names=F)

dim(outTab)

##########

#######cor_all

cor1 = read.table("corResult_TF.txt", header=T,sep="\t",check.names=F)

cor2 = read.table("corResult_GSVA.txt", header=T,sep="\t",check.names=F)

cor3 = read.table("corResult_ssGSEA.txt", header=T,sep="\t",check.names=F)

lnc = intersect(as.vector(cor1[,1]),as.vector(cor2[,1]))

lnc = intersect(lnc,as.vector(cor3[,2]))

write.table(file="final_cor_sig.txt",lnc,sep="\t",quote=F,row.names = F)

cor4=cor1[which(cor1[,1]%in%lnc),]

cor5=cor2[which(cor2[,1]%in%lnc),]

cor6=cor3[which(cor3[,2]%in%lnc),]

cor_all=rbind(cor4,cor5,cor6)

dim(cor4)

dim(cor5)

dim(cor6)

dim(cor_all)

write.table(file="cor_all.txt",cor_all,sep="\t",quote=F,row.names=F)

##########

listgene = read.table("finallistgene.txt", header=F)

listpathway = read.table("finallistpathway.txt", header=F)

listssgsea= read.table("finallistssgsea.txt", header=F)

listgene= as.vector(listgene[,1])

listpathway= as.vector(listpathway[,1])

listssgsea= as.vector(listssgsea[,1])

rt=read.table("Group_symbol_FPKM.txt",sep="\t",header=T,check.names=F)

rt <- as.matrix(rt)

rownames(rt)=rt[,1]

exp=rt[,2:ncol(rt)]

dimnames=list(rownames(exp),colnames(exp))

immuneGene=matrix(as.numeric(as.matrix(exp)),nrow=nrow(exp),dimnames=dimnames)

finalgene = immuneGene[listgene,]

finalpathway = gsva_es[listpathway,]

TIMER2 = read.table("ssgseaOut.txt", row.names=1 ,header=T,sep="\t",check.names=F)

finalssgeas=TIMER2[listssgsea,]

sameSample=intersect(colnames(finalgene),colnames(finalpathway))

sameSample=intersect(sameSample,colnames(finalssgeas))

finalgene= finalgene[,sameSample]

finalpathway= finalpathway[,sameSample]

finalssgeas= finalssgeas[,sameSample]

finalall=rbind(finalgene,finalpathway,finalssgeas)

pdf("corHeatmap_all.pdf",height=10,width=10)

ggcorrplot(corr=cor(t(finalall)),

type = "lower",

lab = T,lab_size = 1.2,tl.cex = 5)

dev.off()

########final_key_list

rt=read.table("limmaTab.xls",sep="\t",header=T,check.names=F,row.names = 1)

diffExp=read.table("Group_symbol_FPKM.txt",sep="\t",header=T,check.names=F)

diffExp <- as.matrix(diffExp)

rownames(diffExp)=diffExp[,1]

exp=diffExp[,2:ncol(diffExp)]

dimnames=list(rownames(exp),colnames(exp))

diffExp=matrix(as.numeric(as.matrix(exp)),nrow=nrow(exp),dimnames=dimnames)

gene=read.table("finallistgene.txt",sep="\t",header=F)

immuneDiffAll=rt[intersect(gene[,1],row.names(rt)),]

immuneDiffGene=intersect(gene[,1],rownames(rt))

hmExp=diffExp[immuneDiffGene,]

immuneDiffResult=immuneDiffAll[immuneDiffGene,]

immuneDiffResult=rbind(ID=colnames(immuneDiffResult),immuneDiffResult)

write.table(immuneDiffResult,file="finallistgene_diff.xls",sep="\t",col.names=F,quote=F)

immuneGeneExp=rbind(ID=colnames(hmExp),hmExp)

write.table(immuneGeneExp,file="finallistgene_geneExp.txt",sep="\t",quote=F,col.names=F)

Type=read.table("clinical.txt",sep="\t",header=T,check.names=F,row.names = 1)

Type=as.data.frame(Type)

plotdata = t(hmExp)

# rainbow是R的一个函数，用于产生彩虹色

E = normalizeBetweenArrays(plotdata, method="quantile")

plotdata = E

pdf(file="heatmap_finallistgene.pdf", width = 8,height =6)

pheatmap(t(plotdata),

annotation=Type,

color =colorRampPalette(c("#3F007D", "white", "#E34A33"))(50),

cluster_cols =F,

show_colnames = F,

show_rownames = T,

fontsize = 12,

fontsize_row=12,

fontsize_col=10)

dev.off()
